# Identification of novel CSF biomarkers for neurodegeneration and their validation by a high-throughput multiplexed targeted proteomic assay

**DOI:** 10.1186/s13024-015-0059-y

**Published:** 2015-12-01

**Authors:** Wendy E. Heywood, Daniela Galimberti, Emily Bliss, Ernestas Sirka, Ross W. Paterson, Nadia K. Magdalinou, Miryam Carecchio, Emma Reid, Amanda Heslegrave, Chiara Fenoglio, Elio Scarpini, Jonathan M. Schott, Nick C. Fox, John Hardy, Kailash Bahtia, Simon Heales, Neil J. Sebire, Henrik Zetterburg, Kevin Mills

**Affiliations:** Centre for Translational Omics, University College London Institute of Child Health, 30 Guilford Street, London, WC1N 1EH UK; Neurology Unit, Department of Pathophysiology and Transplantation, University of Milan, Fondazione Cà Granda, IRCCS Ospedale Policlinico, Via F.Sforza 35, 20122 Milan, Italy; Dementia Research Centre, University College London Institute of Neurology, London, UK; Reta Lila Weston Institute of Neurological Studies, UCL Institute of Neurology, Queen Square, London, UK; Neuropediatrics Unit, IRCCS Istituto Neurologico Carlo Besta, Milan, 20133 Italy; Great Ormond Street Hospital for Children, London, WC1N 3JH UK; Clinical Neurochemistry Laboratory, Institute of Neuroscience and Physiology, Department of Psychiatry and Neurochemistry, The Sahlgrenska Academy, University of Gothenburg, 431 80 Mölndal, Sweden

**Keywords:** Lewy body dementia, Alzheimer’s disease, Targeted proteomics, CSF biomarker

## Abstract

**Background:**

Currently there are no effective treatments for many neurodegenerative diseases. Reliable biomarkers for identifying and stratifying these diseases will be important in the development of future novel therapies. Lewy Body Dementia (LBD) is considered an under diagnosed form of dementia for which markers are needed to discriminate LBD from other forms of dementia such as Alzheimer’s Disease (AD). This work describes a Label-Free proteomic profiling analysis of cerebral spinal fluid (CSF) from non-neurodegenerative controls and patients with LBD. Using this technology we identified several potential novel markers for LBD. These were then combined with other biomarkers from previously published studies, to create a 10 min multiplexed targeted and translational MRM-LC-MS/MS assay. This test was used to validate our new assay in a larger cohort of samples including controls and the other neurodegenerative conditions of Alzheimer’s and Parkinson’s disease (PD).

**Results:**

Thirty eight proteins showed significantly (*p* < 0.05) altered expression in LBD CSF by proteomic profiling. The targeted MRM-LC-MS/MS assay revealed 4 proteins that were specific for the identification of AD from LBD: ectonucleotide pyrophosphatase/phosphodiesterase 2 (*p* < 0.0001), lysosome-associated membrane protein 1 (*p* < 0.0001), pro-orexin (*p* < 0.0017) and transthyretin (*p* < 0.0001). Nineteen proteins were elevated significantly in both AD and LBD versus the control group of which 4 proteins are novel (malate dehydrogenase 1, serum amyloid A4, GM_2−_activator protein, and prosaposin). Protein-DJ1 was only elevated significantly in the PD group and not in either LBD or AD samples. Correlations with Alzheimer-associated amyloid β-42 levels, determined by ELISA, were observed for transthyretin, GM2 activator protein and IGF2 in the AD disease group (r^2^ ≥ 0.39, *p* ≤ 0.012). Cystatin C, ubiquitin and osteopontin showed a strong significant linear relationship (r^2^ ≥ 0.4, *p* ≤ 0.03) with phosphorylated–tau levels in all groups, whilst malate dehydrogenase and apolipoprotein E demonstrated a linear relationship with phosphorylated-tau and total-tau levels in only AD and LBD disease groups.

**Conclusions:**

Using proteomics we have identified several potential and novel markers of neurodegeneration and subsequently validated them using a rapid, multiplexed mass spectral test. This targeted proteomic platform can measure common markers of neurodegeneration that correlate with existing diagnostic makers as well as some that have potential to show changes between AD from LBD.

**Electronic supplementary material:**

The online version of this article (doi:10.1186/s13024-015-0059-y) contains supplementary material, which is available to authorized users.

## Background

**Fig. 1:**
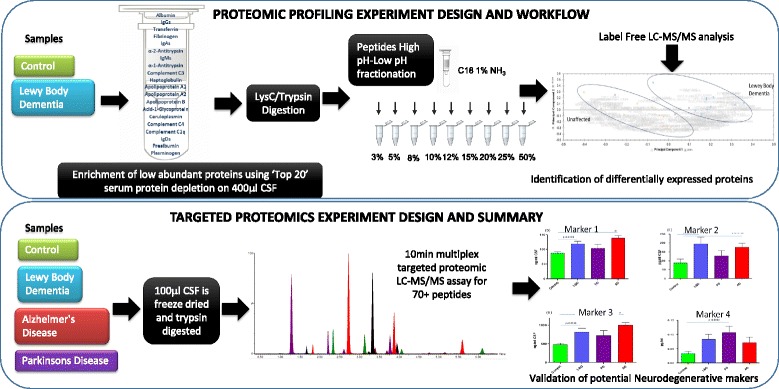
Graphical summary of the work carried out ‘from biomarker discovery to validation’. Proteins found in the proteomic profiling experiment were incorporated into a targeted MRM-LC-MS/MS based assay along with other markers in the literature

The global impact of dementia is increasing rapidly with 150 million people estimated to be affected worldwide by 2015. This is largely due to neurodegenerative disorders such as Alzheimer’s disease (AD) and Lewy body dementia (LBD) [1]. Neurodegenerative diseases, like Parkinson’s disease (PD), are also common and cause significant morbidity. These disorders are all strongly age-related and the expected increase in patients with these long-term conditions presents a huge social and economic challenge. Therefore, there is an urgent need to find treatments that will slow, delay or prevent these diseases. Accurate diagnosis of neurodegenerative disease will be crucial to treatment development and ultimately, to our ability to offer earlier effective therapeutic interventions. Definitive diagnosis in non-genetic neurodegenerative disorders can currently only be made with histopathological confirmation, and usually only at post-mortem examination [2]. In vivo clinical diagnosis of neurodegenerative disorders (AD, LBD etc.) is difficult especially in the earliest stages: specialist centres typically only achieve an accurate pre-mortem diagnosis in 70–90 % of cases [2]. Therefore, there is a pressing need for efficient, cost-effective biomarkers that can help diagnose patients earlier and more accurately. Furthermore there is increasing recognition that the majority of neurodegenerative diseases have long pre-clinical stages. In addition, for treatments to be effective it may be necessary to initiate treatments well before the onset of symptoms and for this to occur a diagnosis will almost certainly rely on biomarkers. The availability of biomarkers for different neurodegenerative conditions with overlapping symptoms but different aetiologies therefore represents a critical issue for the future availability of disease-modifying treatments. The potential utility of cerebrospinal fluid (CSF) biomarkers in neurodegeneration has been shown by the development of β amyloid 1–42 (Aβ42), total tau protein (h-tau), and phosphorylated tau 181 (p-tau) assays; these are now well established as diagnostic biomarkers of AD and have entered routine clinical practice [3]. Lewy body dementia in particular has a much lower incidence than AD with an estimate of 3.5 cases per 100,000 per year [4] and is widely accepted to be highly under diagnosed and is the most frequently misdiagnosed form of dementia. Markers for LBD are greatly needed therefore the profiling part of this study was focused on identifying an LBD CSF protein signature that could then be tested against other neurodegenerative conditions such as AD and PD. Fig. 1 illustrates the experimental design of the 3 stages of this study. Thus, this work set out to i) perform an in-depth biomarker discovery experiment to identify potentially unique CSF protein signatures for neurodegenerative conditions with the focus on LBD ii) combine these new biomarkers, with conventional biomarkers and potential biomarkers described previously in the literature [5–14], into a rapid, high-throughput and multiplexed test iii) validate these new markers alongside previously described biomarkers in a larger multicentre cohort of samples from patients with AD, PD and LBD.

In summary, this work describes a biomarker discovery type experiment using LBD patient CSF which allowed us to identify several proteins not described previously. In addition, we have developed this further into a ‘one-pot’ targeted proteomic translational assay, capable of quantitating 46 potential biomarkers in under 10 min and using only 100 μl of CSF. Using this targeted proteomics method we were able to validate 4 novel markers for LBD and AD from 2 separate dementia centre cohorts.

## Results

### Biomarker discovery (Label Free Quantitative Proteomics)

In total, >1000 proteins were detected in 400 μl of depleted CSF by label free quantitative proteomic analyses. Analysis of the LBD CSF proteome demonstrated 40 proteins were significantly altered with 22 proteins by a factor of 2-fold or more when compared to the control group (Additional file 1: Table S1). Gene Ontology analysis of the’cellular component’ in the LBD CSF revealed the majority were secretory in origin (71 %) but also a significant proportion were cytosolic (21 %) and interestingly 7 % were determined to be involved in cell junctions (Additional file 2: Figure S1). Using Webgestaldt gene analysis tools [15] those genes found to be dysregulated in the LBD group were observed to be involved in ‘wound healing’ ‘defence response’, the ‘inflammatory response’ and ‘regulation of the response to external stimulus’. In addition, ‘negative regulation of blood coagulation / homestasis’ was observed in 12 genes, 6 genes involved in proteoglycan homeostasis, 5 genes involved in platelet degranulation and 6 genes involved in proteolysis. In the molecular function category, 12 genes involved in carbohydrate binding, 3 in phospholipid binding and 44 genes involved in biopeptide production, enzyme activity/regulation, peptidase regulation and inhibition.

### Targeted proteomic MRM LC-MS/MS assay

**Fig. 2:**
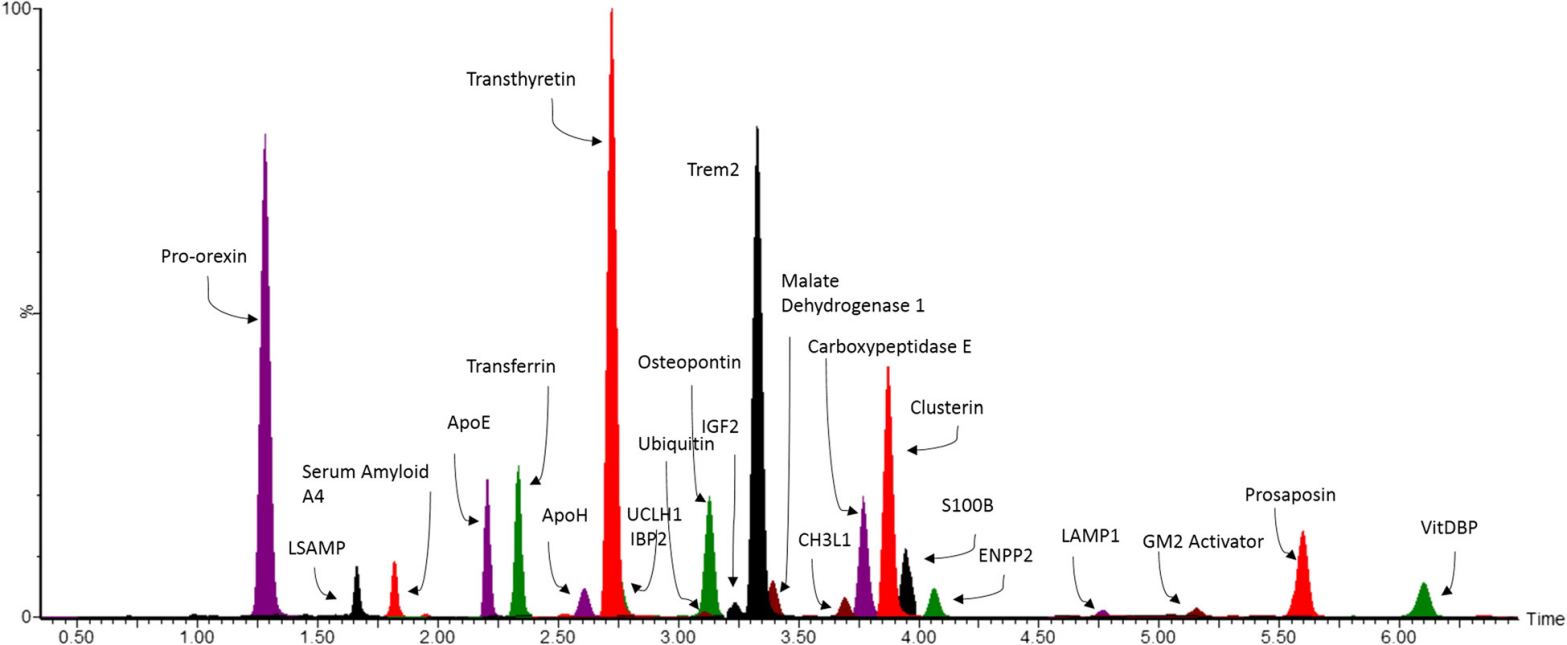
Overlaid chromatogram of the marker peptides included in the multiplexed targeted proteomic assay. The assay was developed to quantitate 74 peptides in a 10 min LC run. Markers significant in the study are shown in the above overlaid chromatogram except for transferrin, serum amyloid A4 and apolipoprotein E which are not shown due to interference from other peaks. All markers are shown individually as endogenous and spiked in Additional file 8: Figure S2

Table 1 summarises all the proteins that were found significantly altered compared to controls in this study. Based on fold-change (>1.5) and quality of the MS data, potential biomarkers identified in the discovery experiments were selected for validation by their development into a multiplexed 10 min, targeted proteomic triple quadrupole, peptide MRM-based assay. Chitinase-3-like protein 1 (YKL-40) and Apolipoprotein E were significant in the label free proteomics analyses but at a cut off of less than 1.5 fold. However as they have been described previously as neurodegeneration markers they were included in the assay to assess them with the other potential markers. Selected AD or PD specific markers described previously in the literature were also included [5–14] in order to compare with LBD. Using timed, or dynamic MRMs, in an UPLC-MS/MS assay, we created a method capable of analysing 74 peptides from 54 proteins using 100 μl of CSF. The full list of biomarkers put forward for validation is given in Additional file 3: Table S2. Out of these 54 potential biomarkers, only 27 resulted in being statistically significant and/or have potential for diagnostic use (Table 1). Fig. 2 includes an overlaid chromatogram of the UPLC-MS/MS chromatogram of the final successful targeted proteomic assay depicting the majority of the proteins/peptide biomarkers.

**Table 1:** Summary of results of potential makers validated using a targeted proteomic mass spectrometry test

Alzheimer’s disease		Lewy body dementia		Parkinson’s disease	
Protein biomarker	Fold-change (statistical significance)	Protein biomarker	Fold-change (statistical significance)	Protein biomarker	Fold-change (statistical significance)
Osteopontin	4.7 (*p* < 0.002)			DJ1	3.1 (*p* < 0.0053)
		Osteopontin	2.3 (*p* < 0.02)	^a^Malate dehydrogenase	2.2 (not significant)
CNDP1	3.2 (*p* < 0.0001)	^a^UCHL1	2.2 (*p* < 0.01)	^a^LSAMP	1.9 (*p* < 0.0136)
^a^Malate dehydrogenase	3.2 (*p* < 0.0001)	Chitinase-3-like protein 1	2.1 (*p* < 0.0004)	Apolipoprotein H	1.8 (*p* < 0.0484)
Apolipoprotein E	2.9 (*p* < 0.0001)	^a^GM_2_ Activator Protein	1.9 (*p* < 0.0087)	Serum Amyloid A4	1.7 (not significant)
Ubiquitin	2.3 (*p* < 0.0015)	^a^Malate dehydrogenase	1.9 (*p* < 0.0082)	Pro-orexin	1.6 (not significant)
^a^GM_2_ Activator Protein	2.3 (*p* < 0.0002)	^a^Serum Amyloid A4	1.9 (*p* < 0.0092)	Osteopontin	1.6 (*p* < 0.02)
IBP2	2.3 (*p* < 0.0001)	Apolipoprotein E	1.7 (*p* < 0.0014)	UCHL1	1.5 (not significant)
^a^Serum Amyloid A4	2.3 (*p* < 0.0007)	CNDP1	1.7 (*p* < 0.0039)	Prosaposin	1.5 (not significant)
^a^Pro-orexin	2.2 (*p* < 0.0017)	Apolipoprotein H	1.7 (*p* < 0.01)	Vitamin D binding protein	1.5 (p = 0.05)
^a^Carboxypeptidase E	2.2 (*p* < 0.0001)	^a^Prosaposin	1.7 (*p* < 0.02)	Chitinase-3-like protein 1	1.4 (not significant)
Apolipoprotein H	2.2 (*p* < 0.0001)	S100B	1.7 (*p* < 0.031)	GM_2_ Activator Protein	1.3 (not significant)
Chitinase-3-like protein 1	2.2 (*p* < 0.0012)	Ubiquitin	1.6 (*p* < 0.011)	S100B	1.3 (not significant)
^a^prosaposin	2.1 (*p* < 0.0001)	Insulin-like growth factor 2	1.6 (*p* < 0.0052)	Apolipoprotein E	1.2 (not significant)
^a^UCHL1	2 (*p* < 0.0108)	Cystatin C	1.6 (*p* < 0.002)	CNDP1	1.2 (not significant)
^a^LAMP1	2 (*p* < 0.0003)	Vitamin D binding protein	1.6 (*p* < 0.01)	Clusterin	1.2 (not significant)
Cystatin C	1.9 (*p* < 0.0001)	IBP2	1.6 (*p* < 0.0052)	Cystatin C	1.2 (not significant)
Vitamin D binding protein	1.9 (*p* < 0.0001)	LAMP1	1.5 (not significant)	Transferrin	1.2 (not significant)
Transthyretin	1.8 (*p* < 0.0001)	^a^Carboxypeptidase E	1.5 (*p* < 0.0097)	LAMP1	1.1 (not significant)
Insulin-like growth factor 2	1.8 (*p* < 0.0015)	^a^TREM 2	1.5 (*p* < 0.0062)	Carboxypeptidase E	1.1 (not significant)
^a^TREM 2	1.8 (*p* < 0.0003)	^a^LSAMP	1.5 (*p* < 0.0004)	TIMP1	1.1 (not significant)
^a^LSAMP	1.8 (0.0009)	Pro-orexin	1.4 (not significant)	Insulin-like growth factor 2	1.1 (not significant)
ENPP2	^a^1.7 (*p* < 0.0001)	Clusterin	1.4 (*p* < 0.0102)	TREM 2	1.1 (not significant)
S100B	1.7 (*p* < 0.0037)	TIMP1	1.4 (*p* < 0.0055)	IBP2	1.1 (not significant)
Clusterin	1.6 (*p* < 0.0001)	Transferrin	1.4 (*p* < 0.01)	Transthyretin	1 (not significant)
Transferrin	1.4 (*p* < 0.0015)	Transthyretin	1.1 (not significant)	Ubiquitin	0.9 (not significant)
TIMP1	1.3 (*p* < 0.018)	ENPP2	1 (not significant)	ENPP2	0.8 (not significant)

The fold-change in the expression of each protein biomarker relative to the control group, are shown in the second column with their p value determined by Mann-Witney *U* test shown in parenthis below it

^a^Denotes new markers not described previously as being potential neurodegenerative markers

### Alzheimer’s disease specific biomarkers

**Fig. 3:**
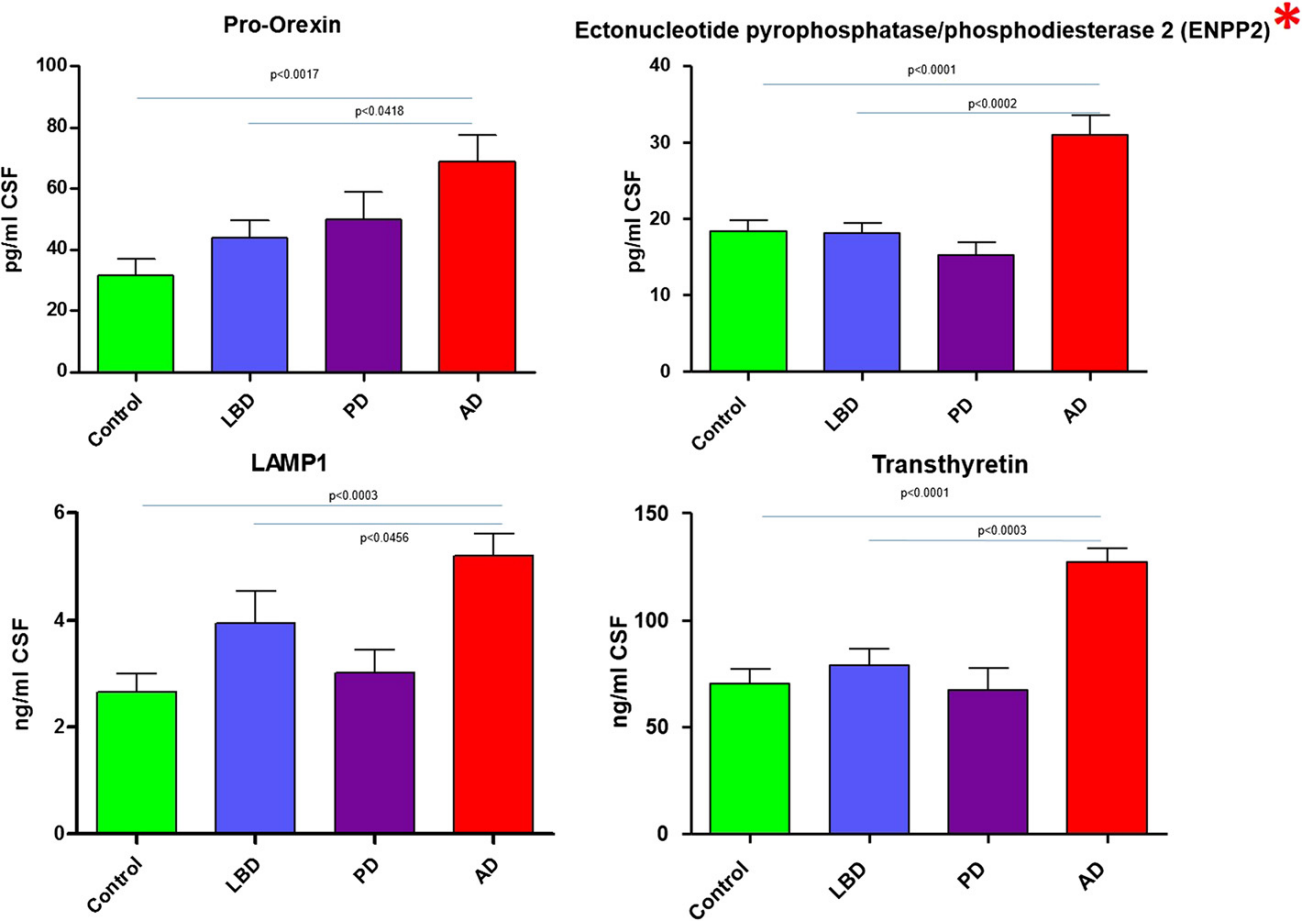
Alzheimer’s disease Specific Markers Graphs showing the results of the multiplexed MRM-based LC-MS/MS assay of protein biomarkers quantitated in the CSF of control. Lewy body dementia (LBD). Parkinson’s disease (PD) and Alzheimer’s disease (AD). All 4 markers show changes in AD specific from other neurodegenerative disease groups and controls. No significant changes are observed in the PD group * Denotes new marker not described previously

The MRM-based mass spectral analysis demonstrated that 4 biomarkers (pro-orexin, LAMP1, transthyretin and ectonucleotide pyrophosphatase/phosphodiesterase 2 (ENPP2/autotaxin)) were statistically significantly elevated in the CSF of AD versus the control cohort (Table 1, Fig. 3) and not in the LBD samples. Two of the four biomarkers have not been described previously as potential diagnostic proteins for Alzheimer’s disease (LAMP1 and ENPP2). ENPP2 was the most statistically significant of all the biomarkers (*p* < 0.0001) and exhibited an approximate 1.7-fold elevation compared to the control cohort. The biomarkers with the greatest fold increase in the AD (Table 1) were osteopontin, carnosine dipeptidase 1 (CNDP1) and malate dehydrogenase, and were approximately 4.7-, 3.4-, 3.2- and 3.2-fold higher in the AD group compared to the control cohort, respectively. However, although these proteins demonstrated the highest-fold mean increase in the AD versus control group, none were disease specific for AD.

### Alzheimer’s disease and Lewy body dementia related biomarkers

**Fig. 4:**
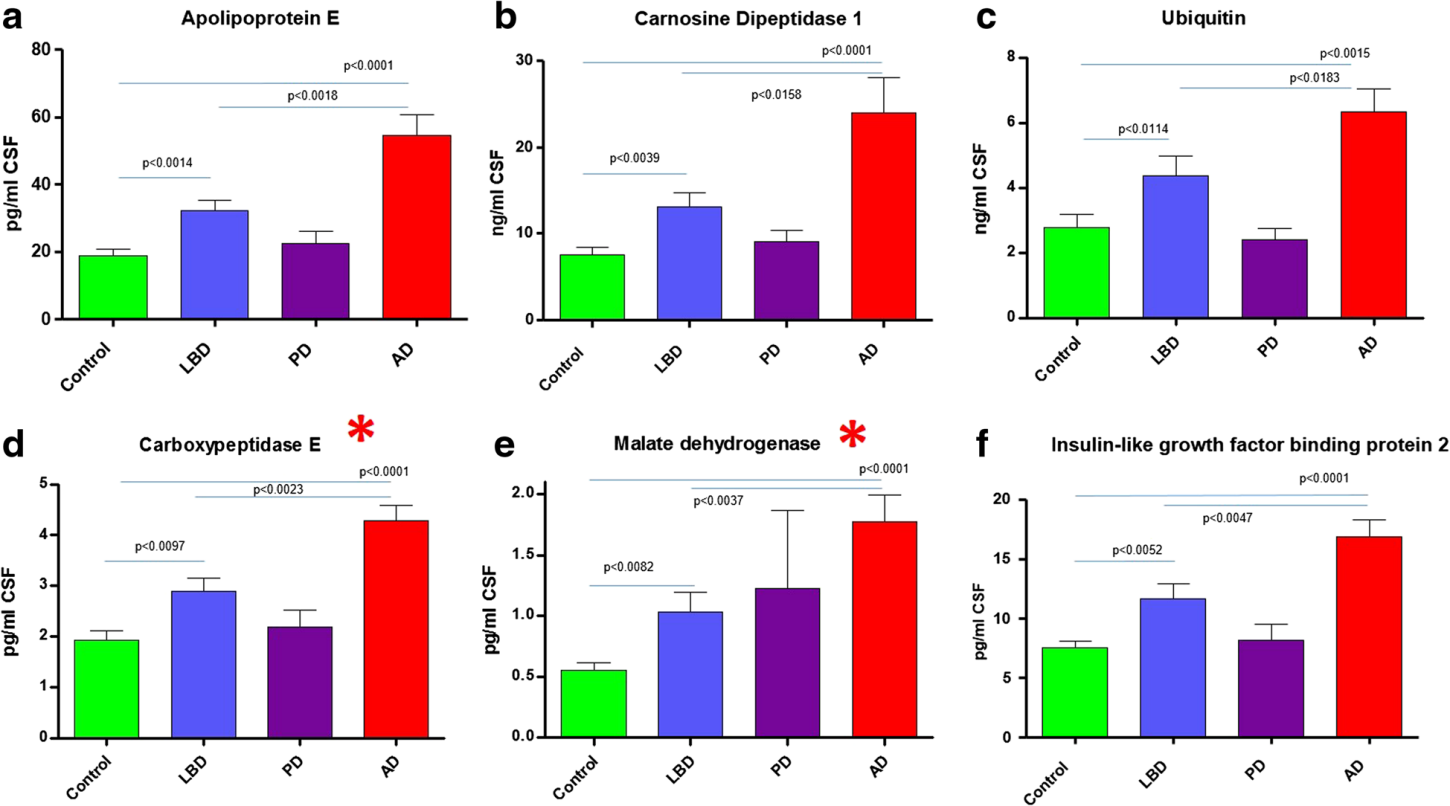
Common dementia markers that are significantly elevated in AD compared to LBD. Graphs **a**-**f** show the results of the targeted proteomic multiplexed assay of protein biomarkers quantitated in the CSF of control, Lewy body dementia, Parkinson’s and Alzheimer’s disease. Graphs **a**-**f** demonstrate the ability of the test to show changes between Lewy Body dementia and Alzheimer’s disease. No significant changes are observed in the PD group.* Denotes new biomarkers not described previously

**Fig. 5:**
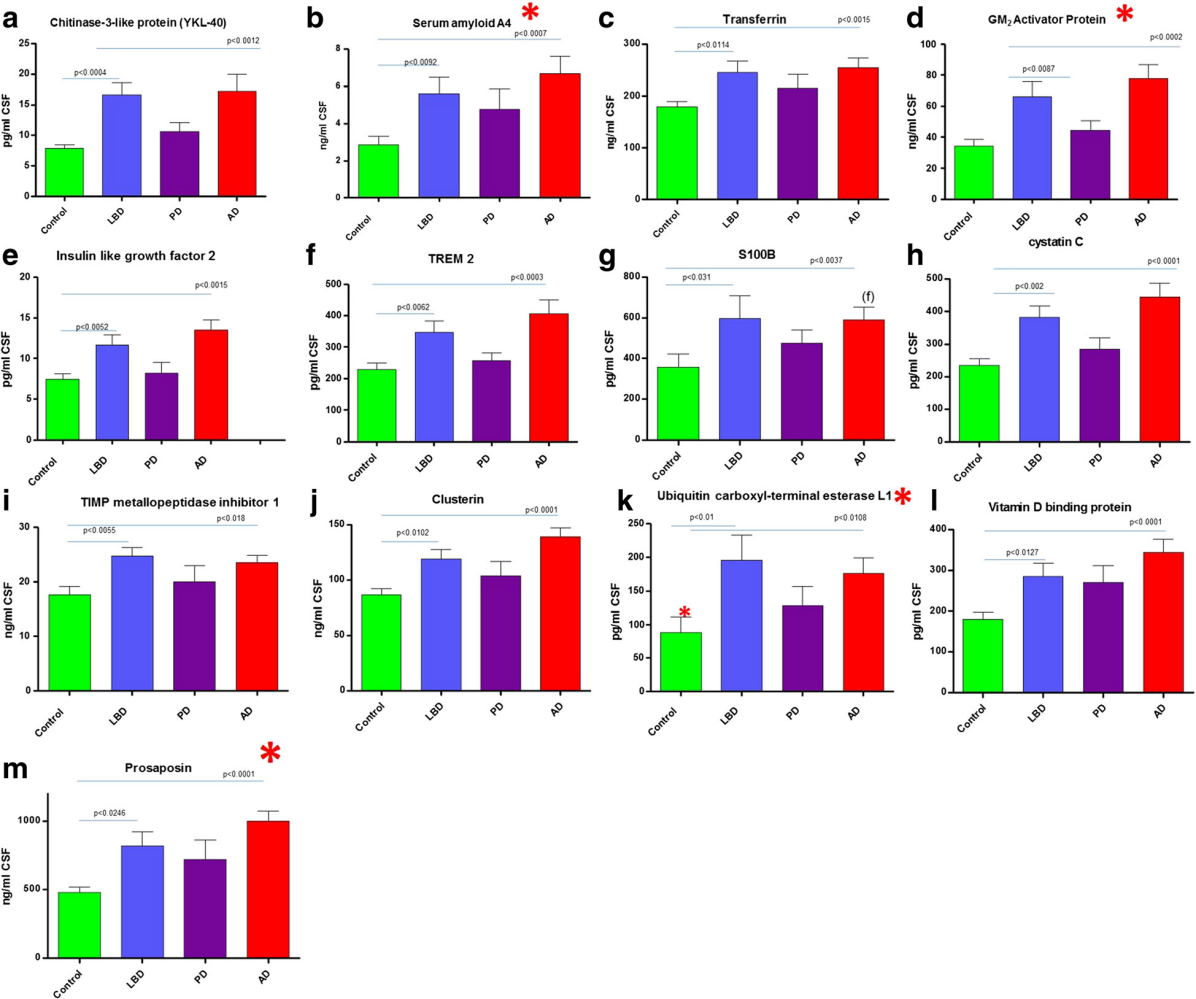
Common dementia markers for LBD and AD. Results of the targeted proteomic multiplexed assay of protein biomarkers quantitated in the CSF. Graphs **a**-**m** show significant changes in Lewy body dementia, and Alzheimer’s disease compared to controls. No significant changes are observed in the PD group * Denotes new biomarkers not described previously

**Fig. 6:**
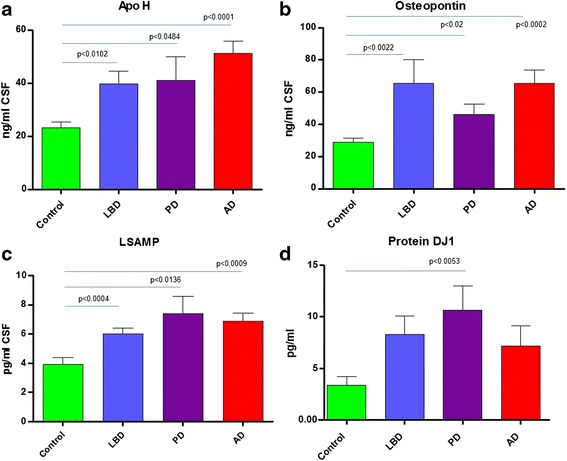
Non-specific markers of neurodegeneration and Protein DJ1 as a marker for Parkinson’s disease. Graphs **a**-**d** show the results of the targeted proteomic multiplexed assay of protein biomarkers quantitated in the CSF of control, Lewy body dementia, Parkinson’s and Alzheimer’s disease. Graphs a-c show biomarkers that show significant changes in all disease groups compared to controls. Protein DJ1 (**d**) shows only a significant change in Parkinson’s disease compared to controls. * Denotes new biomarkers not described previously

A total of 23 protein biomarkers were observed to be statistically significantly elevated in the AD and LBD groups compared to the control group (Table 1, Figs. 4, 5 and 6). Of these 23 proteins, 6 proteins had not been described previously as being potential markers of neurodegeneration in LBD and AD. As described earlier, ENPP2 (autotaxin) and LAMP1, were able to distinguish AD from LBD. Figure 4 shows proteins elevated in both AD and LBD compared to control. These proteins also are even significantly higher in AD compared LBD. Proteins that are also elevated but not statistically significantly between LBD and AD are shown in Fig. 5. These consist of mainly the validated markers in this study and are likely markers of non-specific neurodegeneration. In similarity to the AD specific results, those biomarkers most statistically significantly elevated in the LBD group versus controls, did not demonstrate the greatest fold change in elevation. Those biomarkers showing the greatest fold-change in elevation were osteopontin (2.3-fold), UCHL1 (2.2-fold) and chitinase-3-like protein 1 (2.1-fold, Table 1). Of those biomarkers demonstrating the greatest fold-change in elevation in the LBD group, 5 out of the top 7, had never been described previously as being potential biomarkers for the diagnosis of LBD (UCHL1, GM_2_ activator protein, malate dehydrogenase, serum amyloid A4, Table 1).

### Parkinson’s disease and non-specific neurodegeneration biomarkers

Only one protein, DJ1, was significantly elevated in the Parkinson’s disease group compared to the control cohort (Fig. 6d). DJ1 was the most statistically significant for PD but also the biomarker demonstrating the greatest fold-change in elevation (3.1-fold higher than the mean control concentration, Table 1). However, whilst DJ1 was elevated compared to controls in PD, it was not statistically elevated compared to the LBD and AD groups. In total, only 4 biomarkers were statistically elevated in the PD group versus controls and included DJ1 (*p* < 0.0053), LSAMP (*p* < 0.0136), apolipoprotein H (*p* < 0.0484) and osteopontin (*p* < 0.02,) (Fig. 6).

Three biomarkers were significantly elevated in all disease groups compared to the controls (Fig. 6). These biomarkers were apolipoprotein H, osteopontin and LSAMP. Apolipoprotein H was 1.7-fold (*p* < 0.0014), 1.8-fold (*p* < 0.048) and 2.2-fold (*p* < 0.0001) elevated in the LBD, PD and AD groups, respectively, relative to the control cohort. Similarly, osteopontin was observed to be 2.3-fold (*p* < 0.02), 1.6-fold (*p* < 0.02) and 4.7-fold (*p* < 0.002) elevated in the LBD, PD and AD groups, respectively. Finally, LSAMP also demonstrated significant fold-changes and elevation in all of the neurodegenerative groups of LBD, PD and AD’s relative to the control group of 1.5-fold (*p* < 0.0004), 1.9-fold (*p* < 0.0136) and 1.8-fold (*p* < 0.0009) increases versus the mean of the control group (Table 1, Fig. 6).

### Correlation of existing biomarkers Aβ42 and total and phosphorylated-181 tau with multiplexed test biomarkers

Correlation analyses were performed to compare the relationship between the current biomarkers analysed using the conventionally used ELISA-based methods (total tau (h-tau), p-tau and Aβ42) and the biomarkers developed into the targeted proteomics assay developed in this study. PD samples were not investigated due to the smaller sample number for this group. Correlation analysis was performed on all markers for the control, LBD and AD groups individually with h-tau, p-tau and Aβ42 and results are summarised in Additional file 4: Table S3 and individual graphs are given in Additional file 5: Figure S3, Additional file 6: Figure S4 and Additional file 7: Figure S5.

**Fig. 7:**
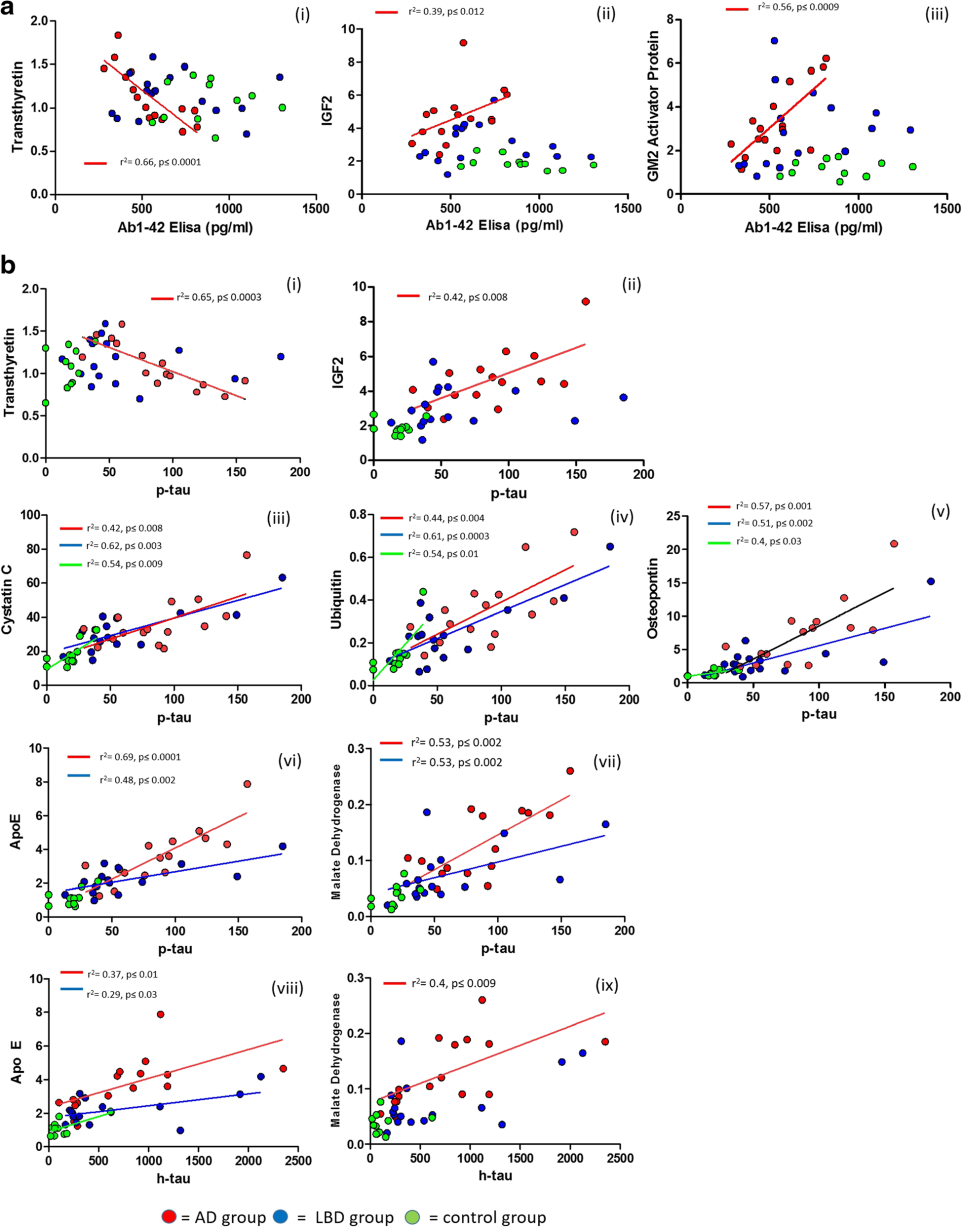
Correlation analysis of multiplexed potential markers with diagnostic ELISA data of the currently used clinical markers. **a** i-iii show markers transthyretin, IGF2 and GM2 activator protein in the AD group which correlate significantly with Aβ1-42 levels (measured by ELISA) in the AD group. There were no correlations observed for any of the markers in the LBD and control groups. **b** i-ii show markers transthyretin and IGF2 which correlate significantly with p-tau (ELISA data) only in the AD group. iii-v show markers cystatin C, ubiquitin and osteopontin which correlate with p-tau in all groups indicating that their expression is likely to reflect p-tau expression. vi and vii show markers ApoE and malate dehydrogenase which correlate with p-tau in both AD and LBD disease groups. viii and ix also show correlation of ApoE and malate dehydrogenase with total tau (h-tau) expression

Transthyretin a marker known to correlate with Aβ42 [16] demonstrated a strong relationship with Aβ42 (Fig. 7a (i)) and p-tau (Fig. 7b (i)) in the AD group but no correlation was observed in the LBD group. This was also noted for IGF2 although the correlation with Aβ42 was weaker (Fig. 7a (ii)). Other strongly associated markers with Aβ42 in AD were GM2 activator protein, chitinase-3-like protein 1 and cystatin C (Additional file 5: Figure S3 and Additional file 4: Table S3).

Three markers cystatin C, osteopontin and ubiquitin showed either a strong or weak association of p-tau in the control group as well as both LBD and AD groups indicating a specific association of these proteins with p-tau levels. CNDP1, apoE and malate dehydrogenase demonstrated strong association of p-tau in both LBD and AD but not in control (Fig. 7b). ApoE and malate dehydrogenase also showed a significant correlation with total tau levels as well as p-tau. Many of the markers did not show any correlation with Aβ42, p-tau and total tau therefore their association with AD and LBD is likely independent from the Aβ42 and tau pathology.

## Discussion

There is a growing need for new and more specific biomarkers in neurodegenerative diseases. The development of new tests will allow us to diagnose these conditions more specifically, and preferentially pre-symptomatically, if we are going to develop new therapies and test their efficacy. For this to occur, there is a need for a more efficient process of taking any potential biomarkers discovered in hypothesis generating experiments and developing them into translational tests suitable for rapid clinical evaluation. We believe this work describes an effective and streamlined process of improving the bottle neck of validating and getting new potential biomarkers to the clinic.

**Fig. 8:**
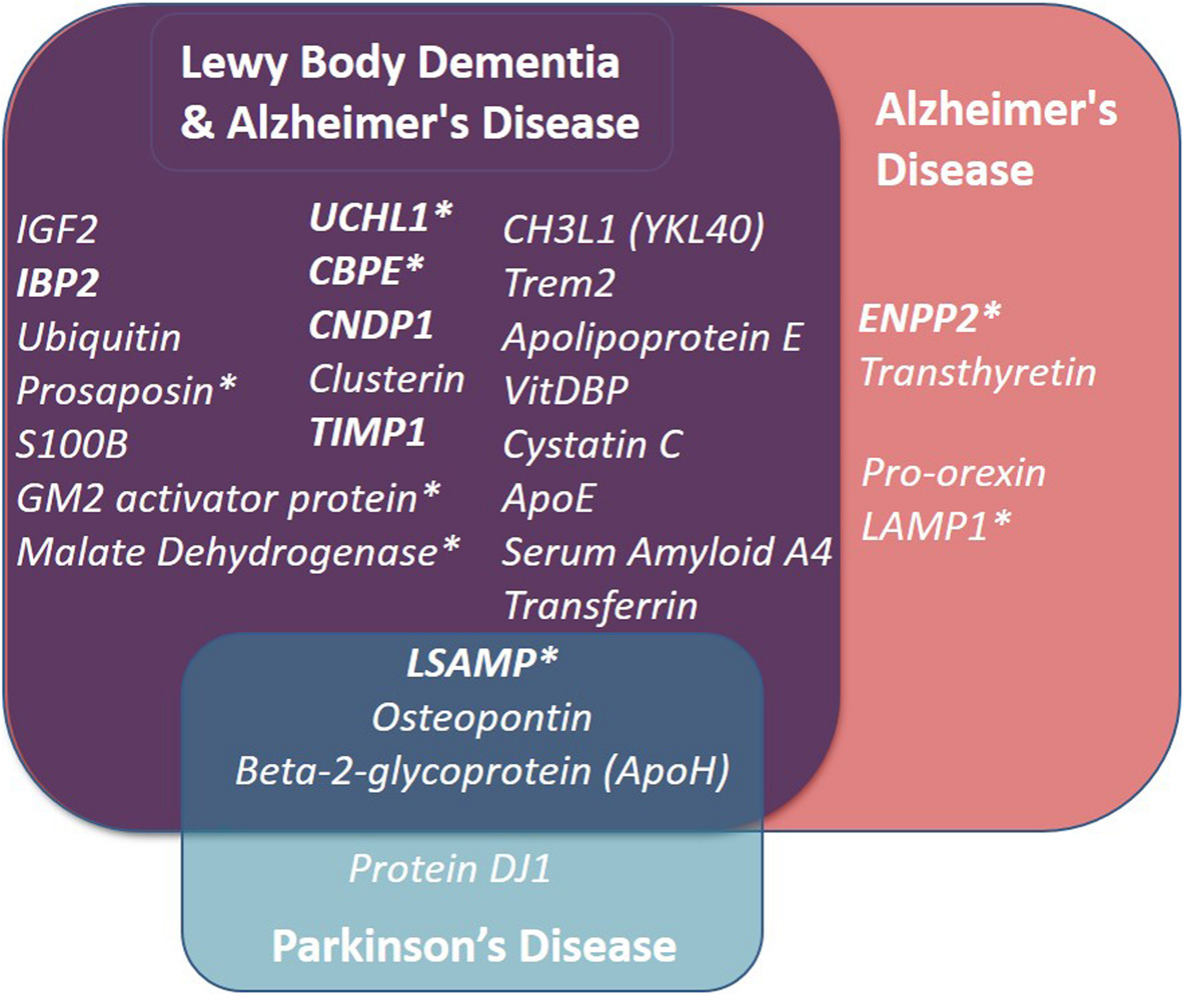
Summarised Venn diagram of the targeted proteomics analysis of the multicentre cohort 2 (AD, LBD, PD and controls). The majority of markers are dementia specific (common to both LBD and AD). However, although there are no markers specific to LBD, the proteins ENPP2, transthyretin, pro-orexin and LAMP1 were specific for the AD group. Three markers were elevated in all groups and Protein DJ was specific to PD. Markers in bold are those discovered in the proteomic profiling experiment. Those with an * are novel markers not previously described

Markers for LBD are in need due to the difficulty in discriminating LBD from AD clinically. Using Label Free quantitative proteomics on LBD CSF we were able to identify several biochemical processes and pathways that were disrupted or perturbed in LBD CSF. Proteins with the potential for diagnostic use were developed into a 10 min, multiplexed, targeted MRM-LC-MS/MS test using a triple quadrupole mass spectrometry based platform (UPLC-MS/MS). The rationale behind the basing of translational tests onto a UPLC-MS/MS as a platform, is that this multiplex targeted proteomic methodology has been proven to be previously beneficial in dementia research [17, 18]. Targeted MRM based proteomic tests are gaining interest in the clinical research field [19, 20] due to its potential to measure multiple markers in one assay and does not have the cost or problems often encountered with the use of antibodies [19, 21]. The assay described in this study had been developed to streamline this method considerably creating a single a ‘one pot’ test with a 10 min LC gradient, with consequent high throughput capability and potential for clinical translation. We validated the test and markers on a larger multicentre cohort of samples including AD, LBD and controls. We were also able to include a cohort of PD CSF samples to compare with the groups and identify markers for overall neurodegeneration. Lumbar puncture is not part of the typical clinical workup for the diagnosis of PD and is usually carried out on research purposes only, hence samples are harder to obtain but were included to highlight the importance of these results in comparison with the other neurodegenerative diseases. Fig. 8 summaries all the CSF biomarkers that were specific for each neurodegenerative condition and those common to each. Only 4 biomarkers detected were specific for AD (Fig. 3) with ENPP2 (autotaxin) having not been described previously as a potential CSF marker of neurodegeneration. Using genetic studies, ENPP2/autotaxin has been described by Umemura et al. [22] as being expressed differentially in the frontal cortex of Alzheimer-type dementia (ATD) patients compared with those of non-AD controls but not the actual protein levels in brain or CSF. ENPP2/autotaxin is a lysophospholipase that acts on lysophosphatidylcholine converting it into lysophosphatidic acid (LPA). LPA is a potent mitogen and has been described previously as having a potential contributory role in AD pathology including Aβ42 formation [23] increased Tau-phosphorylation and neurite retraction in neuronal cells [24]. Indeed Hwang et al. have shown that LPA metabolism maybe a potential target for AD therapy by the use of gintonin as a LPA receptor activating ligand. Gintonin, an extract from ginseng, was shown to attenuate amyloid plaque formation and memory impairment in a transgenic mouse model of AD [25]. The other AD specific markers orexin, and LAMP1 have been either described previously or postulated as being potential CSF biomarkers of AD and thus provide confirmation and validation of the methodology developed in this work [10, 26]. We also included the recently described AD related microglia and macrophage membrane-bound receptor TREM2. Several TREM2 mutations have been identified recently that increase the risk of AD, frontotemporal dementia, PD, and amyotrophic lateral sclerosis [27]. Whilst a lot of research has been performed on the effect of TREM2 variants little has been done to investigate the normal function of TREM2. This study shows that CSF levels of TREM2 are increased in LBD and AD. CSF TREM2 has not been described elevated in dementia. However it has been described in the brain pathology of AD patients [28] and in the CSF of patients with multiple sclerosis and CNS inflammation [29]. This indicates that TREM2 may be an overall neuro-inflammatory marker. ENPP2 (autotaxin) and transthyretin were the most statistically significantly elevated biomarkers (Table 1) and provided complete discrimination between the AD and LBD cohorts. The use of these two biomarkers may provide significant diagnostic value for help in deciding treatment regimens by allowing better discrimination of AD from LBD. Transthyretin has been shown previously to be present in amyloid plaques and our data confirms previous findings of a correlation with Aβ42 levels [16]. However transthyretin is a protein known to be susceptible to post-translational modifications due its free thiol group (particularly in CSF) [30]. This makes reliable measurement of intact transthyretin using antibodies a potential problem and may be a reason as to why some studies have reported reduced levels of transthyretin in AD CSF [16, 31] whilst our study and another targeted proteomic study [17] have reported increased levels. Apolipoprotein E has been studied as a potential biomarker previously in dementia but due to only small changes or ambiguity between studies, the levels of apolipoprotein E have not been considered diagnostically useful. The differences in methodologies between mass spec based assays and immunoassays may explain why our study finds higher levels of apolipoprotein E in dementia compared to others [32, 33].

In addition to identifying 4 potential AD specific biomarkers, a further 19 biomarkers demonstrated a statistically significant elevation in the LBD and AD cohorts versus the control groups. Of these 19 biomarkers, carboxypeptidase E, malate dehydrogenase, serum amyloid A4, GM_2_ activator protein, UCHL1 and prosaposin, had never been described previously as potential CSF biomarkers for neurodegeneration. The proteins prosaposin and GM_2_ activator protein are both chaperone proteins critical for the lysosomal catabolism of glycosphingolipids. A deficiency in GM_2_ Activator Protein has also been associated with the neurological conditions of Tay Sachs and Sandhoff disease. Although, the reason for their elevation in the CSF of LBD and AD patients is unclear, it is probably as a result of generalised lysosomal dysfunction as both these proteins have been detected by the authors in the urine of many different lysosomal storage diseases [34]. The proteins carboxypeptidase E and UCHL1 are both processing enzymes involved in the correct production of neuropeptides/neurohormones and degradation of ubiquitin monomers in the proteasome, respectively. UCHL1 is highly specific to neurons and its expression has been associated with AD, though it has also been shown to interact with α-synuclein another protein implicated in the pathophysiology of PD. Although our data did not show a significant elevation of UCHL1 in the CSF of PD patients compared to the controls.

Little is known about the protein serum amyloid A4 other than it is from the serum amyloid A class of proteins which are a family of apolipoproteins which are known to be involved in the acute inflammatory response. A chronic inflammatory status of neurons is a known factor associated with neurodegeneration but the increased levels of serum amyloid A4, a protein synthesised mainly in the liver and adipocytes is unclear. Finally, malate dehydrogenase was found to be elevated in LBD, PD and AD groups though only statistically significantly elevated in the LBD and AD groups. The role of cytosolic malate dehydrogenase 1 and its elevation in neurodegeneration is unclear as it is an enzyme involved in many metabolic pathways and catalyses the oxidation of malate to oxaloacetate using NAD+. Malate dehydrogenase is an important protein in the citric acid cycle and has a prominent role in gluconeogenesis to create glucose molecules from smaller intermediates. It is known that most neurodegenerative conditions have a mitochondrial component, whether primary or secondary is unclear. The elevation of malate dehydrogenase in the CSF may also be a general marker of mitochondrial dysfunction in its role in the shuttling of malate between the inner mitochondrial membrane and cystosol. Although malate dehydrogenase was >2-fold elevated in the PD group it was not statistically significantly elevated. Only the protein DJ1, also known as PARK7, and a peptidase that is known to have protective role for neurons against cell death, was found to be both >3-fold increased and statistically significantly elevated in the PD group. DJ1 has been shown to be raised in CSF of PD patients previously but the levels were not significant compared with LBD and AD samples and the usefulness of this protein as a diagnostic maker is still to be confirmed [11, 35]. This study shows DJ1 can be measured by targeted proteomics and potentially incorporated into a multiplex test. α-Synuclein was included in the targeted assay but it was not significantly altered in any of the groups tested using this method, which is in agreement with earlier studies showing only small or no changes in the CSF levels of the protein when measured using immunochemical techniques in synucleinopathies like LBD and PD [36].

The correlation analysis of the markers with the established markers given in Additional file 5: Figure S3, Additional file 6: Figure S4, Additional file 7: Figure S5 and Fig. 7 gives an insight into the changes occurring from control to disease and indicate the pathology behind the markers. No correlation with any of the markers were observed for the LBD group. Only specific associations could be observed in the AD group for Aβ42. Transthyretin has been shown previously to have a linear relationship with Aβ42 CSF levels [16]. Our data did demonstrate a correlation just with AD and not with LBD and controls (Fig. 7i) confirming this relationship. Transthyretin also revealed a strong linear relationship with p-tau and a weak relationship with total tau levels. IGF2 also showed an AD specific relationship with Aβ42 and p-tau levels. Many of the markers revealed correlations with both AD and LBD (Additional file 4: Table S3) for p-tau and total tau. Those with the strongest correlations were CNDP1, apoE, malate dehydrogenase, osteopontin, cystatin C and ubiquitin. The latter three also showed association of p-tau levels in the control group. This indicates these markers are more directly associated with p-tau and may only reflect p-tau levels in CSF and therefore have limited use diagnostically. Many markers did not show a relationship with either Aβ42, p-tau and total tau levels and are likely unrelated to the direct pathology of Aβ42 and tau plaques in AD and LBD. These markers may indicate other processes ongoing in neurodegeneration and warrant further investigation.

One limitation of this study is that some patients with LBD pathology can also exhibit amyloid plaques [33]. As with other CSF dementia biomarker studies we acknowledge that we cannot exclude these comorbidities at this point. Also although there were no statistically significant differences between age of patients and controls, we acknowledge that age ranges between patients and controls are heterogeneous, and patients are older than controls. Nevertheless, controls have been followed up for 1 to 4 years and so far none of them developed dementia. In addition, CSF amyloid, total tau and p-tau levels in these subjects are within the normal values (Table 2), suggesting that neither AD-related pathogenic events nor neurodegenerative ones are occurring.

**Table 2:** Mean and ± SEM sample data for the two centre cohorts used for the biomarker discovery and validation cohorts

Biomarker discovery cohort	Control *n* = 15	LBD *n* = 10		
Gender (M:F)	5:10	6:4		
Mean Age at Sampling (yrs ± SD)	61 ± 9.9	71.9 ± 4.3		
Median disease duration (yrs ± SD)	n/a	3.4 ± 1.57		
Mean Aβ42 levels (pg/ml ± SD)	905.41 ± 237	695.6 ± 330.7		
Mean h-tau levels (pg/ml ± SD)	136.58 ± 162	523 ± 432		
Mean p-tau181 levels (pg/ml ± SD)	19.33 ± 11.11	52 ± 39		
Validation cohort	Control *n* = 15	LBD *n* = 17	PD *n* = 7	AD *n* = 16
Gender (M:F)	5:10	10:7	5:2	6:10
Mean Age at Sampling (yrs ± SD)	61 ± 9.9	72.1 ± 5.5	68.1 ± 3.4	78.1 ± 6.67
Median disease duration (yrs ± SD)		3.3 ± 1.9	4.1 ± 2.78	
Mean Aβ42 levels (pg/ml ± SD)	905.41 ± 237	663.5 ± 268	886.4 ± 232	503.68 ± 165.88
Mean h-tau levels (pg/ml ± SD)	136.58 ± 162	679 ± 673	139 ± 93	733.77 ± 481.25
Mean p-tau levels (pg/ml ± SD)	19.33 ± 11.11	64.5 ± 48.8	26 ± 11	93.38 ± 31.55

Control, LBD, PD and 7 AD samples were received from centre 1 and 9 AD samples from centre 2. As the two centres share the same clinical workup for AD the data in Table 2 has been combined

## Conclusions

Multiplexed targeted proteomics is a useful technique for streamlining biomarker validation. We have validated markers for neurodegeneration (summary Fig. 8) that are shared between Alzheimer’s disease, Lewy body dementia and Parkinson’s disease. Also markers for dementia shared between Alzheimer’s disease and Lewy body dementia. We have also identified and validated Alzheimer’s disease specific markers ectonucleotide pyrophosphatase/phosphodiesterase 2, lysosome-associated membrane protein 1, pro-orexin and transthyretin that have the potential to discriminate Lewy body dementia from Alzheimer’s disease. These markers can provide the clinician with further information towards an improved diagnosis and also with further future stratification pave the way for monitoring of disease progression for assessment of future potential treatments. However as with all biomarker discoveries markers will need to be further validated in a larger cohort of samples, and with the levels of the biomarkers stratified with such parameters as disease severity, age of onset and age after diagnosis.

## Methods

### CSF sample criteria, collection and routine analysis

Control CSF samples are challenging to obtain and the samples used in this study have been selected to age match as closely as possible to the disease cohorts (Table 2). Multiple correlation analysis of the markers was performed on the control samples (51–79 years) and only markers that did not show any significant change with age are presented in this study.

The experimental design is summarised in Fig. 1. Two sample cohorts, a biomarker discovery cohort (cohort 1, *n* = 28) and a validation cohort (cohort 2, *n* = 55), were obtained from two separate centres (University of Milan, Ospedale Policlinico, Milan, Italy and University of Gothenburg, Sweden) sharing the same clinical workup. Sample data is given in Table 2. Regarding patients with AD, all patients had altered CSF Aβ42, total tau and p-tau levels, thus confirming the clinical diagnosis [37] with an accuracy of about 90 %, in accordance with more recent criteria [38, 39]. LBD diagnosis was made according to criteria published by McKeith et al. [40]. PD was diagnosed according to current criteria [41]. Controls consisted of patients undergoing CSF analysis for non-neurodegenerative conditions.

Ethics, consent and permissions: For centre 1 Informed consent to participate in this study was given by all subjects or their caregivers. Centre 2: The study procedure has been approved by the ethical committees in Gothenburg. All participants gave informed consent to participate in research.

Clinical CSF was sampled according to a standard protocol at both centres [42]. CSF samples were obtained in polypropylene tubes by LP at the L4/L5 or L3/L4 interspace, centrifuged at 4 °C and stored at ≤ −30 °C until analysis. CSF cell counts, glucose and proteins were determined. Routine analysis to exclude damage of the Blood Brain Barrier (BBB) included measurement of albumin by rate nephelometryand the intrathecal IgG production. The albumin quotient (CSF albumin/serum albumin) X 103 and the IgG index (CSF albumin/serum albumin)/(CSF IgG/serum IgG) were calculated and samples without BBB damage were used in the study which confirm that the proteins measured in this study are produced intrathecally and not leaking from the periphery [43].

### Aβ42, h-tau, p-tau 181 measurement

CSF total tau (h-tau) concentration was determined using a sandwich enzyme-linked immunosorbent assay (ELISA) (INNOTEST hTAU-Ag, Fujirebio, Ghent, Belgium) specifically constructed to measure all tau isoforms irrespectively of phosphorylation status, as previously described [44]. CSF p-tau181 was measured using a sandwich ELISA specifically detecting tau phosphorylated at amino acid 181 (INNOTEST PHOSPHO-TAU (181P), Fujirebio, Ghent, Belgium). Aβ42 levels were determined using a sandwich ELISA (INNOTEST ß- AMYLOID [1–42], Fujirebio, Ghent, Belgium) [45]. Optimal cutoffs for identifying AD were as follows: Aβ42 < 550 pg/mL; h-tau > 400 pg/mL and p-tau > 80 pg/mL [46].

### Biomarker discovery: label-free proteomic analyses (2D-LC-MS^e^)

Four hundred microliters of CSF was enriched for low abundant proteins using a ‘Top20’ serum protein depletion column as shown in Fig. 1 (Sigma, UK). Depleted CSF was precipitated with 3 volumes of ice cold acetone and double digested with Lys C and trypsin proteases (Sigma, UK) as described previously [34]. Peptides were offline fractionated using the high pH fractionation technique [47]. Yeast enolase peptide standard was added and fractions analysed using label free quantitation on a Waters QToF Premier mass spectrometer coupled to a NanoAquity liquid chromatography system (Waters, Manchester). Ten fractions were analysed using a 1.5 h LC-MS^e^ analyses as described previously in our laboratory [48]. Proteins were identified using Waters ProteinLynx Global server v 2.5 and a UniProt human reference proteome database to which the sequence of P00924 yeast enolase and P00761 porcine trypsin were added manually. Fixed modifications of carbamidomethylation of cysteines, dynamic modifications of deamidation of asparagine/glutamine and oxidation of methionine, up to 3 missed cleavage sites and maximum protein mass 800 kDa. False discovery rate was set at 4 %, mass tolerance for ion and fragments were set to auto. Only proteins with >95 % confidence were exported for differential expression analysis using Progenesis LC-MS (non-linear dynamics) software.

### Targeted proteomics: MRM-based triple quadrupole mass spectral assay

Candidate markers were selected based on significance, fold change and quality of the label free proteomics analysis data. Additional and potential neurodegenerative biomarkers determined from the literature were also included in the final multiplexed assay (see Table S2). Representative quantotypic peptides for each protein were determined from the label free proteomics data (top 3 most abundant, optimum daughter spectra for quantitation). Other peptides were selected using the open source online global proteome machine MRM database at www.thegpm.org [49]. Bioactive peptides were designed by using either the N or C-terminus part of the peptide to specify the native peptide from precursor molecules. Custom synthesised peptides (Genscript, USA) were used to optimise the peptide detection and determine the retention time and identify unequivocally the correct peak/s in CSF (individual chromatograms provided in Additional file 2: Figure S1). Two transitions were selected for each peptide. Clean transitions without interfering peaks were used. Twenty nanograms of yeast enolase protein standard (Sigma, UK) and 10–50 pmols heavy labelled peptide standards (Thermo Scientific, UK) were added to 100 μl of CSF. CSF was freeze dried and trypsin digested as described previously [50]. A single 35 μl injection of each CSF digest was injected onto a Waters CORTECS UPLC C18 + Column, 90 Å, 1.6 μm, 3 mm × 100 mm column attached to a C18+ VanGuard pre-column. UPLC and MS tune conditions were performed as described previously [34]. Dynamic Multiple Reaction Monitoring was performed over a 10 min gradient with a minimum of 0.01 s dwell time for quantitative transitions and minimum 12 data points per peak on Waters Xevo TQ-S MS. QC runs of pooled CSF digests were run in triplicate at the start of the run and then every 10 injections. A CV within + /- 10 % for each QC was considered acceptable. CSF was spiked with peptides to create standards with average concentrations of biomarker levels and analysed for intra- and inter-batch variation. Chromatograms were analysed using Waters Targetlynx software. Peptides were standardised by either using a spiked heavy labelled peptide or to a yeast enolase peptide. Absolute levels were obtained from standard curves. Standard curve linearity of r^2^ > 0.9 was achieved for all calibration curves (see Additional file 8: Figure S2). Data was exported to excel and GraphPad Prism for statistical analysis. Intra-batch variation was determined as being between 3.0 and 5.1 % and inter-batch variation being 7.6–8.5 % (*n* = 10, 3 consecutive days). A standard curve 0–40 pmols / 100 μl CSF of each peptide was analysed at the start and the end of the run for quantitation and performance standardisation (cv <10 % was considered acceptable).

### Statistical analysis

Analyses included data QC for peptide performance (coefficient of variance), QC of sample preparation and LC-MS/MS performance (yeast enolase), group comparisons (linear regression, non-parametric Mann Witney test). Pearson correlation analysis was used only when the data met parametric assumptions. In all other instances non-parametric version, Spearman correlation, was used and significant linearity determined by the F-test. Group age ranges were checked for significant difference using a Kruskal-Wallis with Dunns post-test of which no significant difference was confirmed.

## Additional files

Additional file 1:
**Table S1.** Protein identification data with greater or equal to 95 % confidence were exported from Waters ProteinLynx Global Server v 2.5 to Non-linear dynamics Progenesis LC-MS for differential expression analysis. (XLSX 18 kb) Additional file 2:
**Figure S1.** Gene ontology analysis of CSF from patients using the web-based Gene Set Analysis Toolkit (WebGestaldt). (PPTX 364 kb) Additional file 3:
**Table S2.** List of peptides and transitions included in Multiplex targeted proteomics assay. (XLSX 20 kb) Additional file 4:
**Table S3.** Summary of pearson correlation coefficient r2 and p values for each group with each conventional CSF marker. (XLSX 13 kb) Additional file 5:
**Figure S3.** Individual correlation graphs for aβ42 marker in each condition. R^2^ and p values are given in supplementary table 3. Red boxed graphs have *p* < 0.01 yellow squared p value 0.05-0.01. (PPTX 654 kb) Additional file 6:
**Figure S4.** Individual correlation graphs for p-tau in each condition. R^2^ and p values are given in supplementary table 3. Red boxed graphs have *p* < 0.01 yellow squared p value 0.05-0.01. (PPTX 3872 kb) Additional file 7:
**Figure S5.** Individual correlation graphs for total tau in each condition. R^2^ and p values are given in supplementary table 3. Red boxed graphs have *p* < 0.01 yellow squared p value 0.05-0.01. (PPTX 623 kb) Additional file 8:
**Figure S2.** Top chromatograms are taken from a patient CSF sample bottom chromatograms are CSF spiked peptides. (PPTX 1279 kb)

## Abbreviations

CSF: Cerebrospinal fluid
LBD: Lewy body dementia
AD: Alzheimer’s disease
PD: Parkinson’s disease
ENPP2: Ectonucleotide pyrophosphatase/phosphodiesterase family member 2
LAMP1: Lysosome-associated membrane glycoprotein 1
IBP2: Insulin-like growth factor-binding protein 2
IGF2: Insulin-like growth factor 2
TREM2: Triggering receptor expressed on myeloid cells 2
TIMP1: Metalloproteinase inhibitor 1
UCHL1: Ubiquitin carboxyl-terminal hydrolase isozyme L1
VDBP: vitamin D binding protein
LSAMP: Limbic system-associated membrane protein
MRM: Multiple reaction monitoring
p-tau: phosphorylated tau
h-tau: Total tau
