# Erratum to: Identification of novel CSF biomarkers for neurodegeneration and their validation by a high-throughput multiplexed targeted proteomic assay

**DOI:** 10.1186/s13024-016-0086-3

**Published:** 2016-02-23

**Authors:** Wendy E. Heywood, Daniela Galimberti, Emily Bliss, Ernestas Sirka, Ross W. Paterson, Nadia K. Magdalinou, Miryam Carecchio, Emma Reid, Amanda Heslegrave, Chiara Fenoglio, Elio Scarpini, Jonathan M. Schott, Nick C. Fox, John Hardy, Kailash P. Bhatia, Simon Heales, Neil J. Sebire, Henrik Zetterberg, Kevin Mills

**Affiliations:** Centre for Translational Omics, University College London Institute of Child Health, 30 Guilford Street, London, WC1N 1EH UK; Neurology Unit, Department of Pathophysiology and Transplantation, University of Milan, Fondazione Cà Granda, IRCCS Ospedale Policlinico, Via F.Sforza 35, 20122 Milan, Italy; Dementia Research Centre, University College London Institute of Neurology, London, UK; Reta Lila Weston Institute of Neurological Studies, UCL Institute of Neurology, Queen Square, London, UK; Neuropediatrics Unit, IRCCS Istituto Neurologico Carlo Besta, Milan, 20133 Italy; Great Ormond Street Hospital for Children, London, WC1N 3JH UK; Clinical Neurochemistry Laboratory, Institute of Neuroscience and Physiology, Department of Psychiatry and Neurochemistry, The Sahlgrenska Academy, University of Gothenburg, 431 80 Mölndal, Sweden

After publication of this work [1], we noted that the names of two authors Kailash Bhatia and Henrik Zetterberg had been misspelled. The names have now been corrected above.

## References

[1] Heywood WE, Galimberti D, Bliss E, Sirka E, Paterson RW, Magdalinou NK (2015). Identification of novel CSF biomarkers for neurodegeneration and their validation by a high-throughput multiplexed targeted proteomic assay. Mol Neurodegener.

