# Supplementary material for: Identification of novel CSF biomarkers for neurodegeneration and their validation by a high-throughput multiplexed targeted proteomic assay
Source: Mol Neurodegener. 2015 Dec 1;10:64. doi: 10.1186/s13024-015-0059-y (PMC4666172; doi:10.1186/s13024-015-0059-y)
Supplement: Additional file 2: — Figure S1. Gene ontology analysis of CSF from patients using the web-based Gene Set Analysis Toolkit (WebGestaldt). (PPTX 364 kb) [file 13024_2015_59_MOESM2_ESM.pptx]

## Slide 1
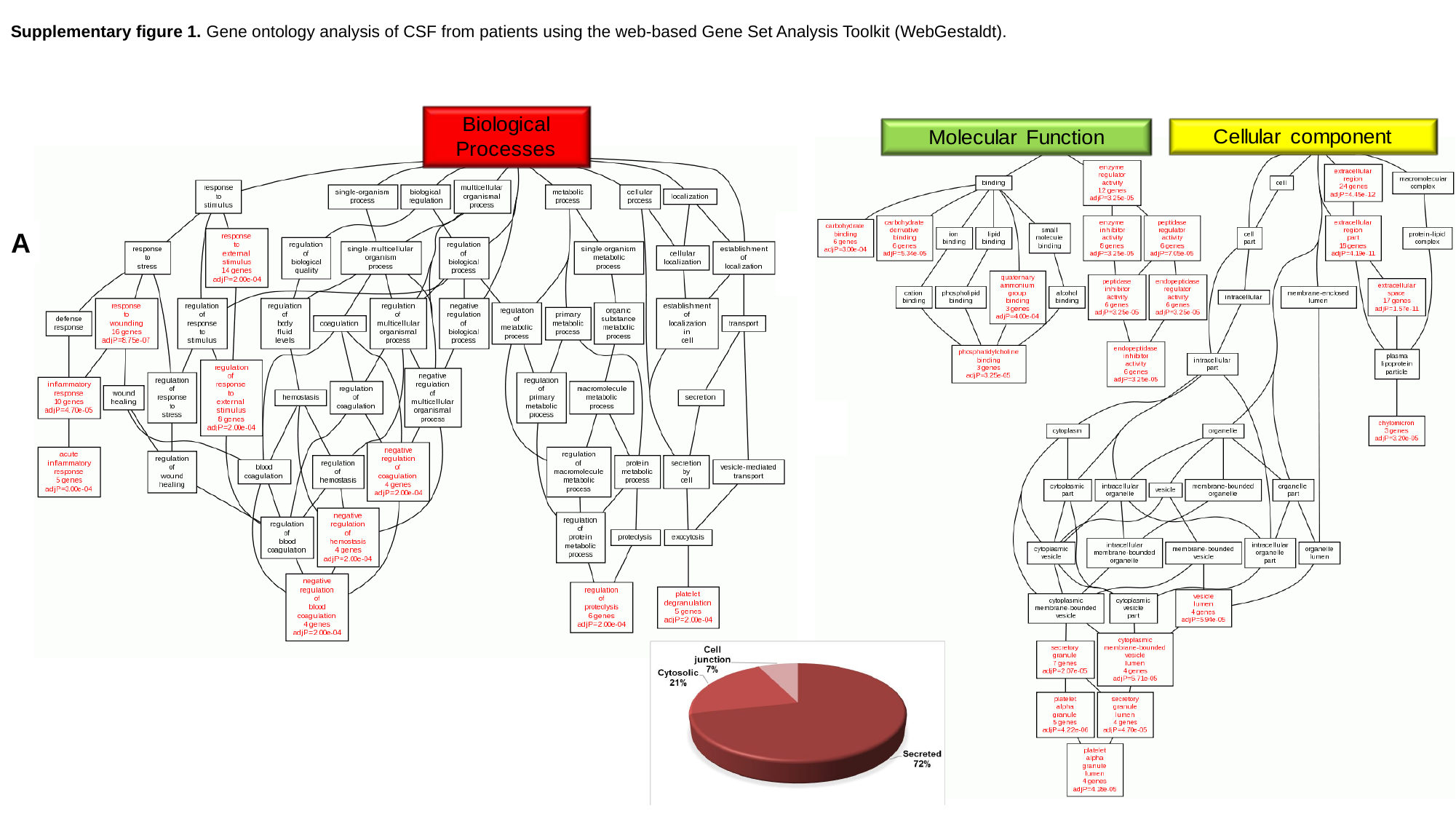

Supplementary figure 1. Gene ontology analysis of CSF from patients using the web-based Gene Set Analysis Toolkit (WebGestaldt).
A
Figure 2
