# Supplementary material for: Identification of novel CSF biomarkers for neurodegeneration and their validation by a high-throughput multiplexed targeted proteomic assay
Source: Mol Neurodegener. 2015 Dec 1;10:64. doi: 10.1186/s13024-015-0059-y (PMC4666172; doi:10.1186/s13024-015-0059-y)
Supplement: Additional file 7: — Figure S5. Individual correlation graphs for total tau in each condition. R2 and p values are given in supplementary table 3. Red boxed graphs have p < 0.01 yellow squared p value 0.05-0.01. (PPTX 623 kb) [file 13024_2015_59_MOESM7_ESM.pptx]

## Slide 1
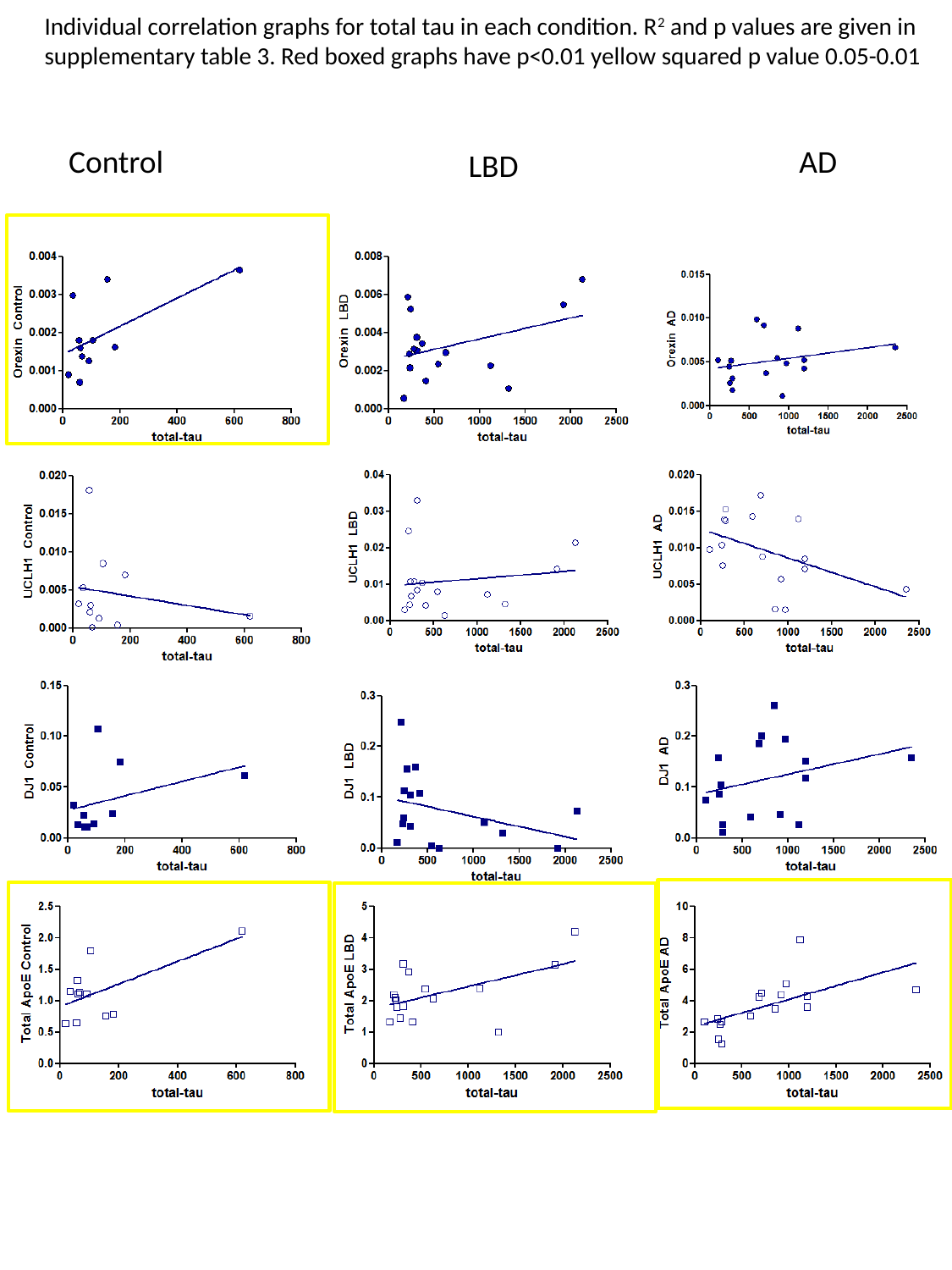

Individual correlation graphs for total tau in each condition. R2 and p values are given in supplementary table 3. Red boxed graphs have p<0.01 yellow squared p value 0.05-0.01
Control
AD
LBD

## Slide 2
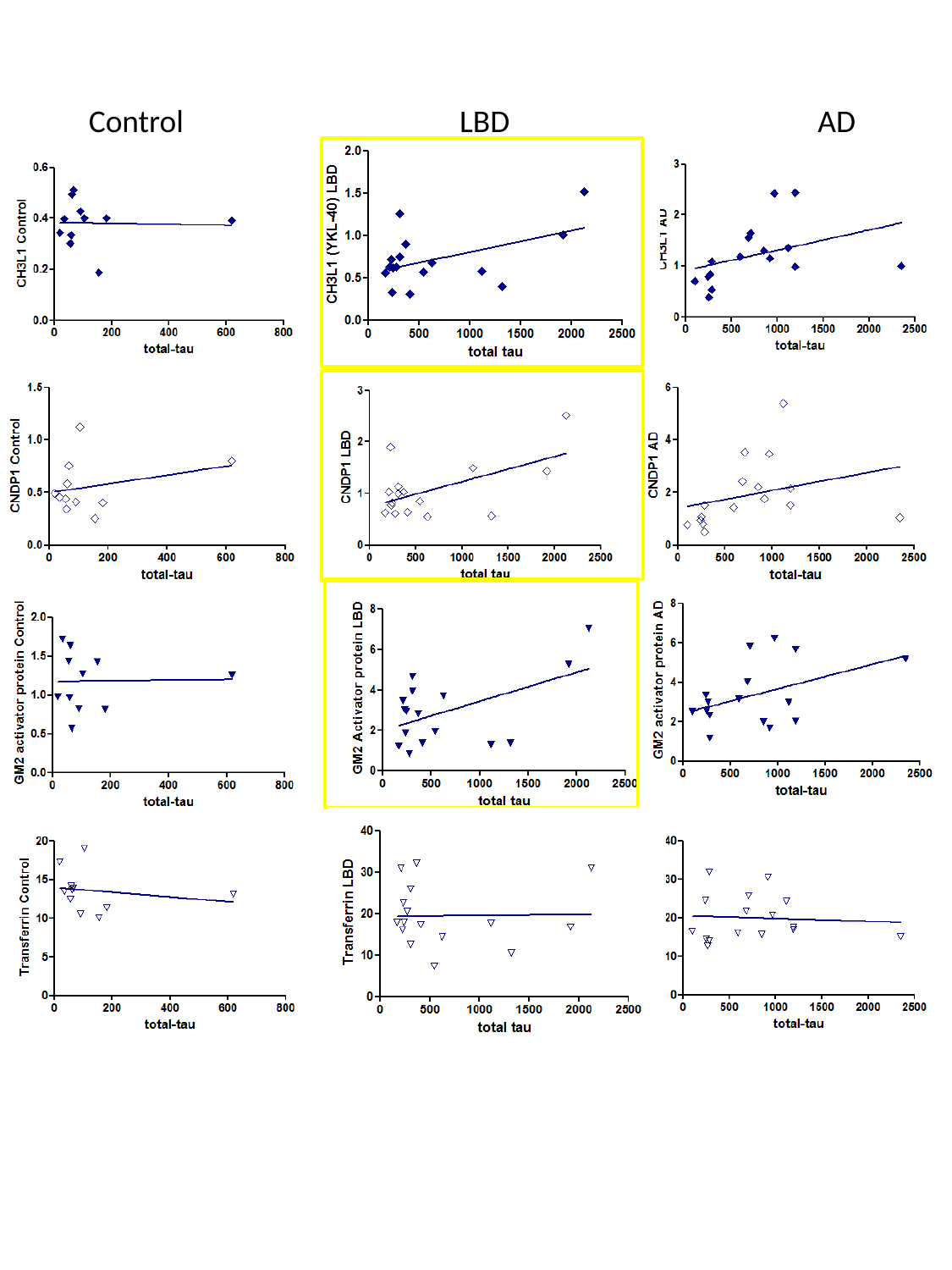

Control
LBD
AD

## Slide 3
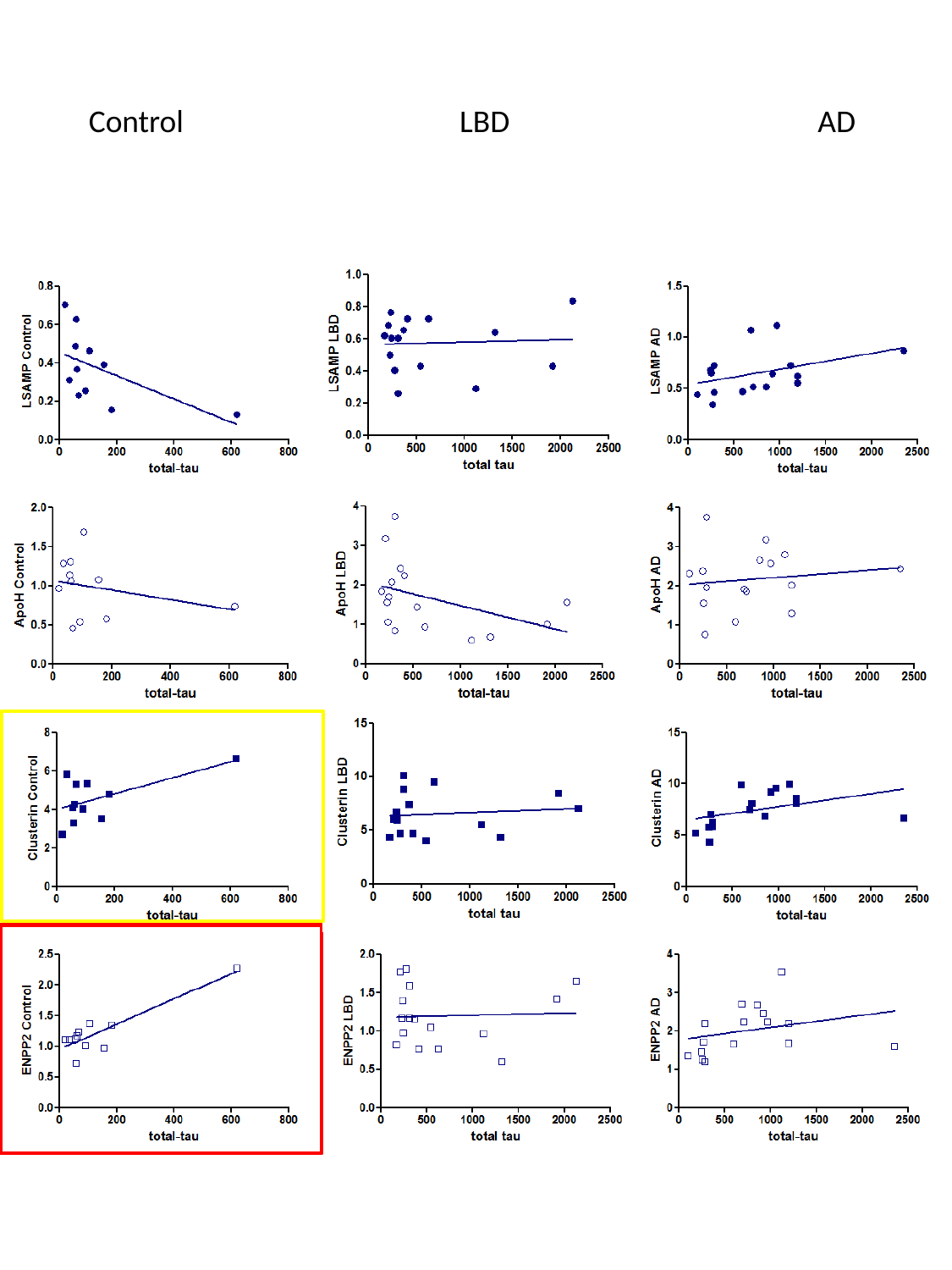

Control
LBD
AD

## Slide 4
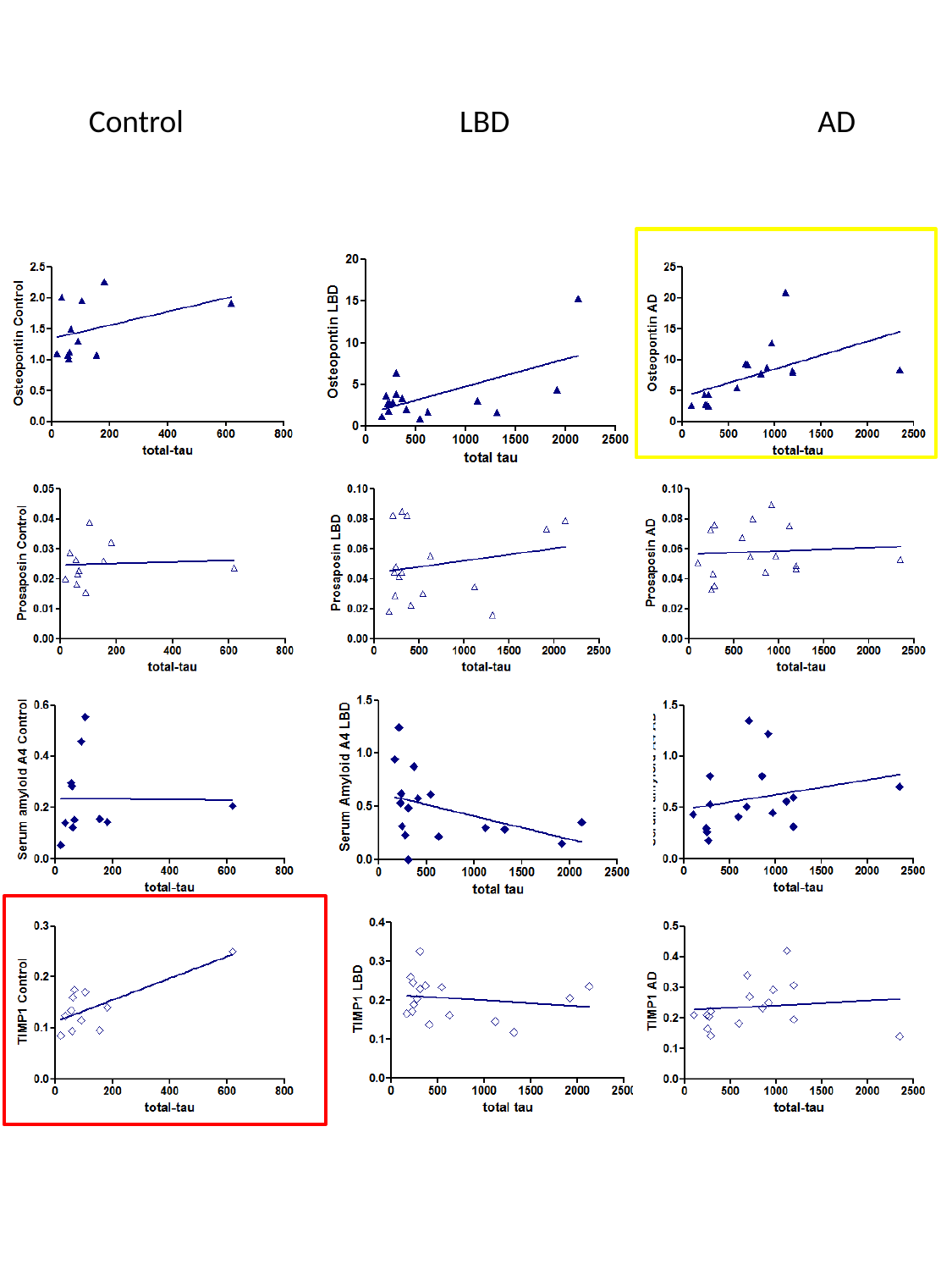

Control
LBD
AD

## Slide 5
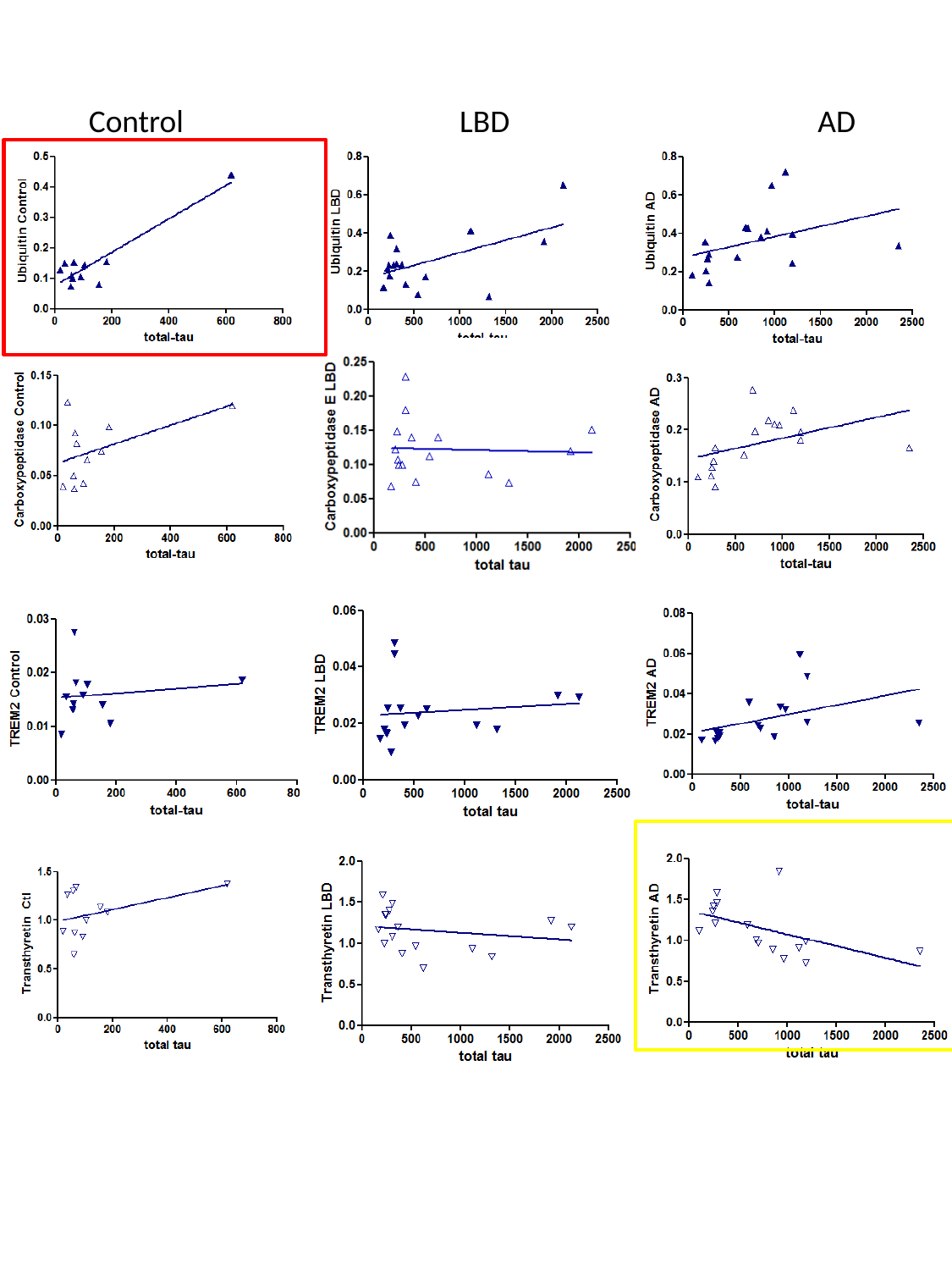

Control
LBD
AD

## Slide 6
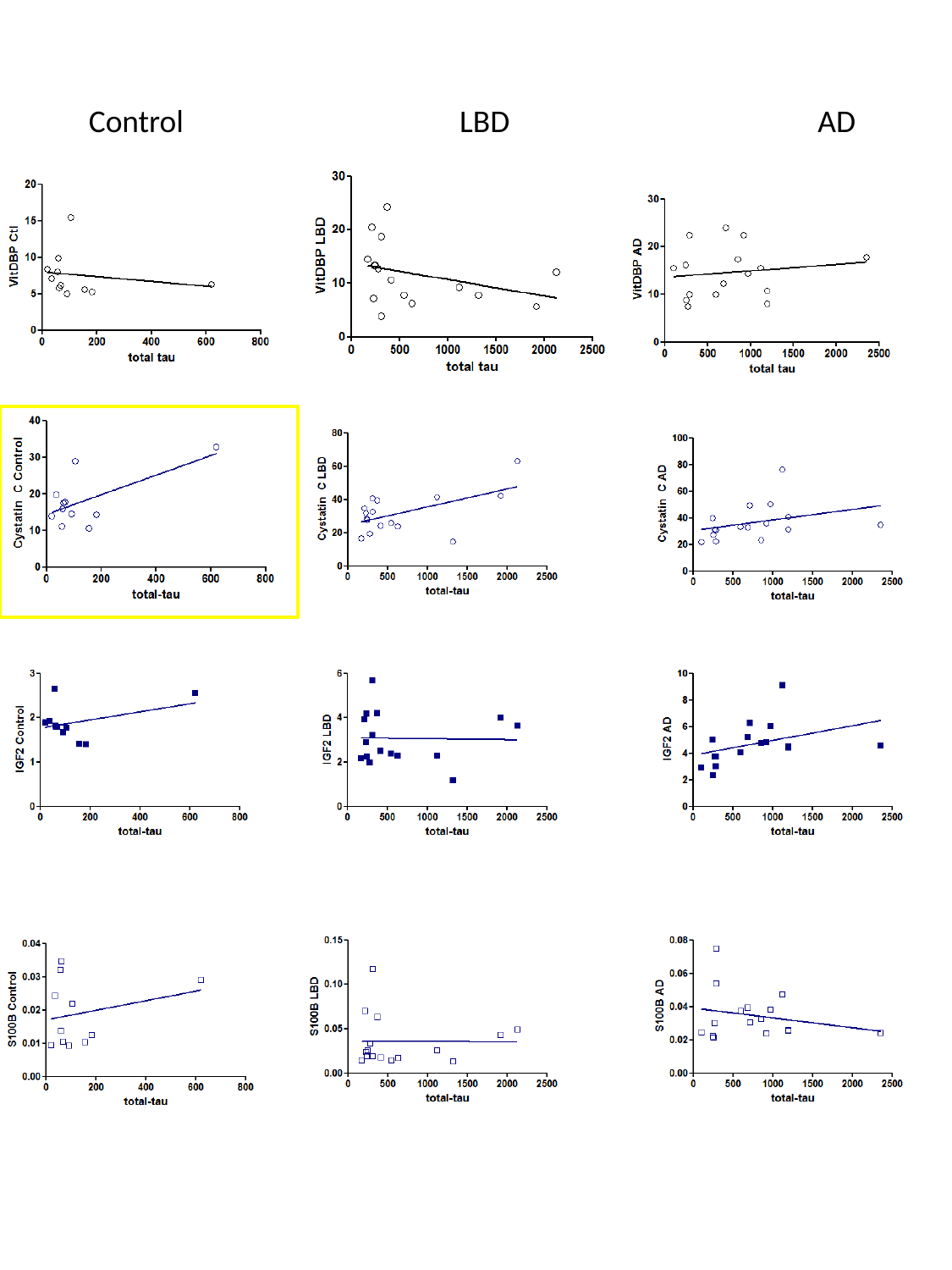

Control
LBD
AD

## Slide 7
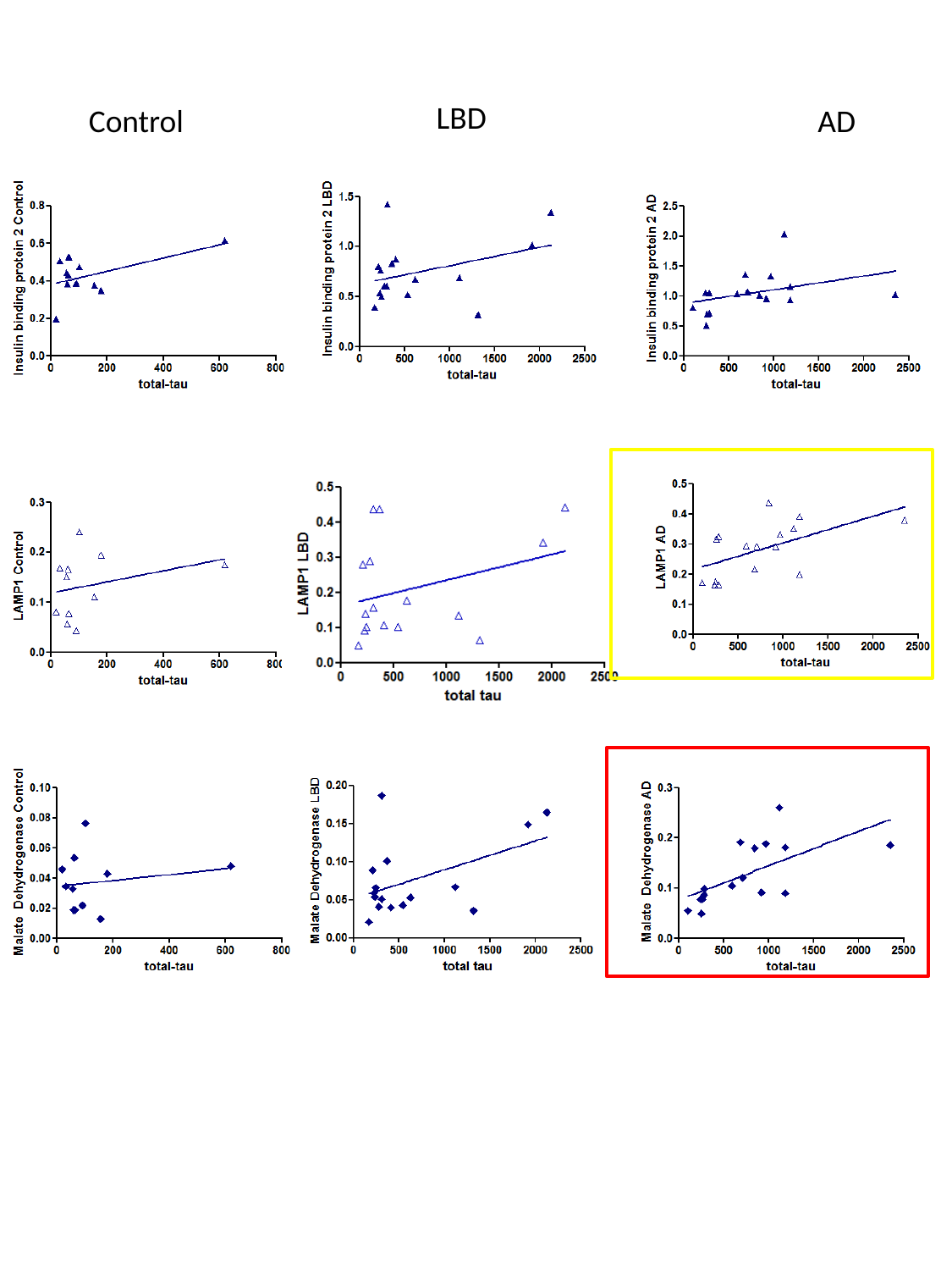

LBD
Control
AD
