# Supplementary material for: Identification of novel CSF biomarkers for neurodegeneration and their validation by a high-throughput multiplexed targeted proteomic assay
Source: Mol Neurodegener. 2015 Dec 1;10:64. doi: 10.1186/s13024-015-0059-y (PMC4666172; doi:10.1186/s13024-015-0059-y)
Supplement: Additional file 8: — Figure S2. Top chromatograms are taken from a patient CSF sample bottom chromatograms are CSF spiked peptides. (PPTX 1279 kb) [file 13024_2015_59_MOESM8_ESM.pptx]

## Slide 1
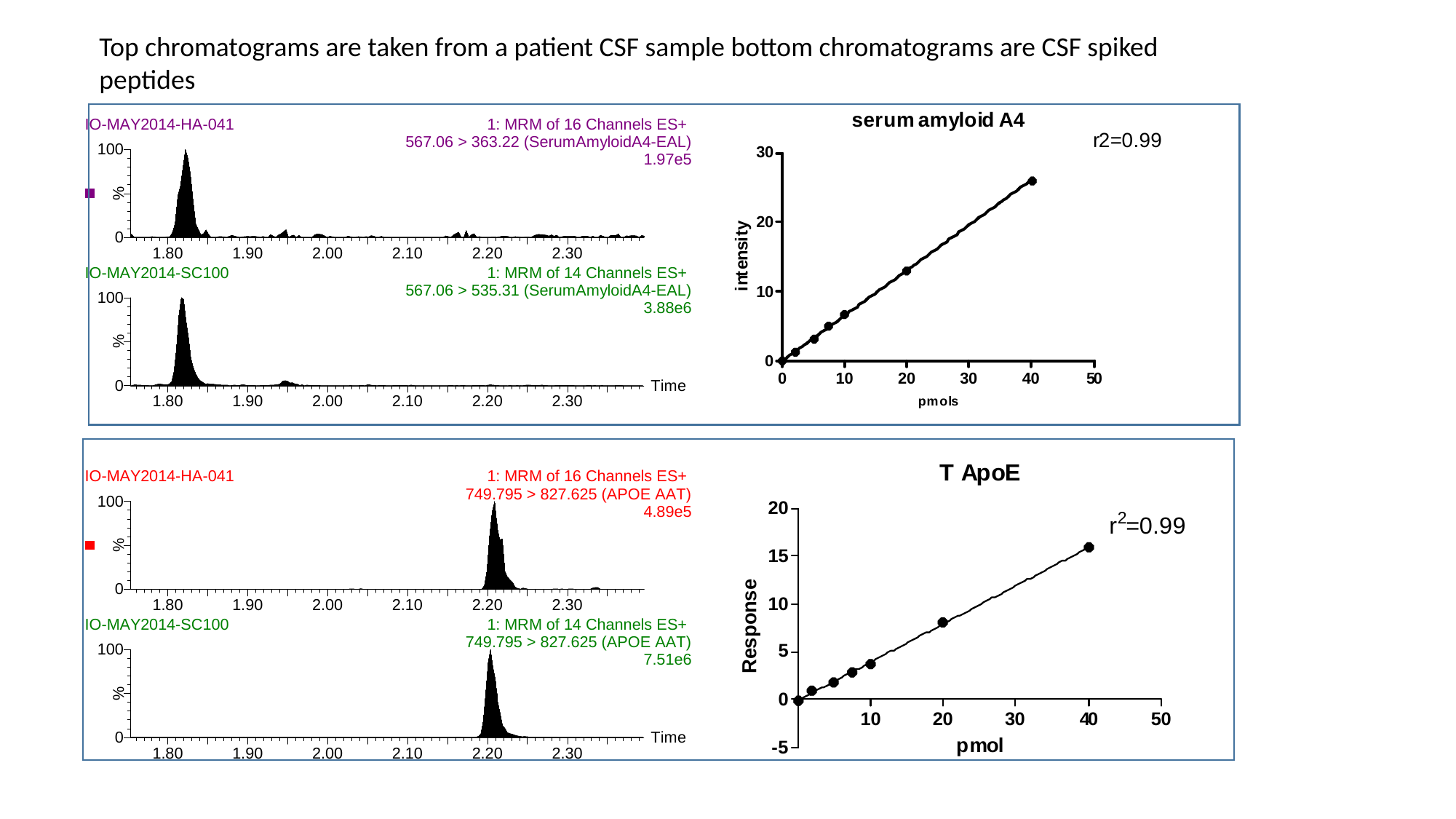

Top chromatograms are taken from a patient CSF sample bottom chromatograms are CSF spiked peptides

## Slide 2
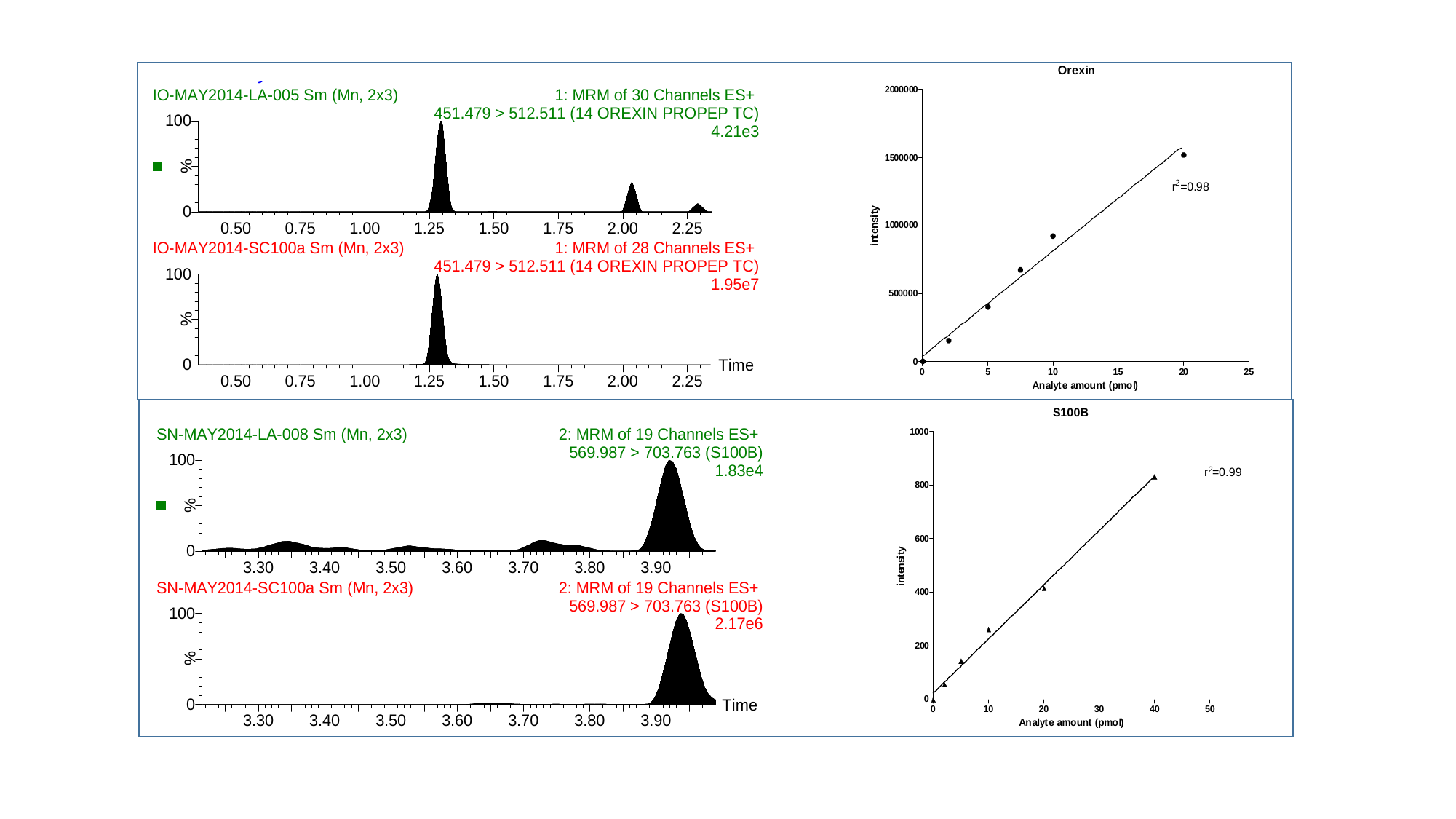

## Slide 3
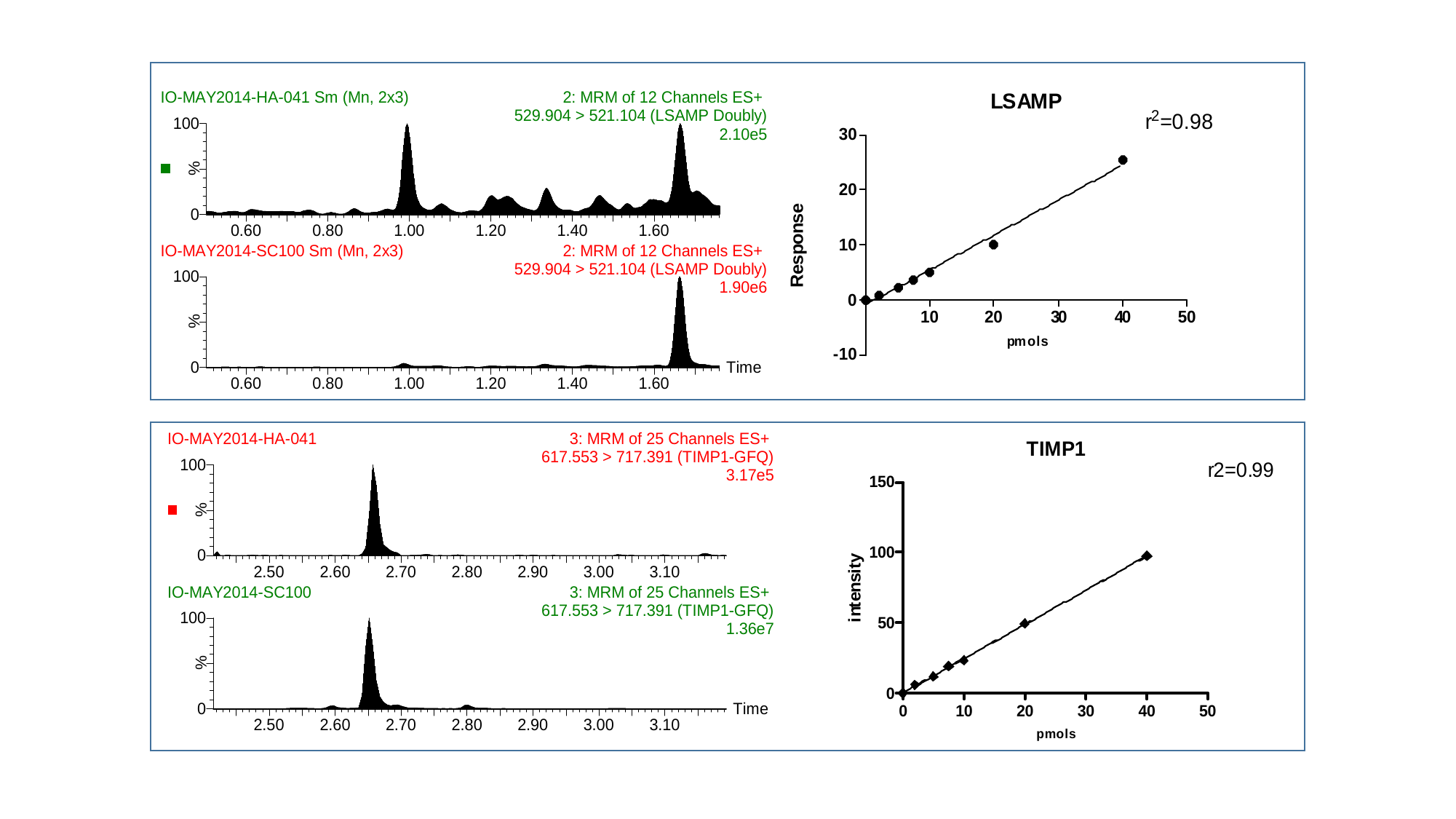

## Slide 4
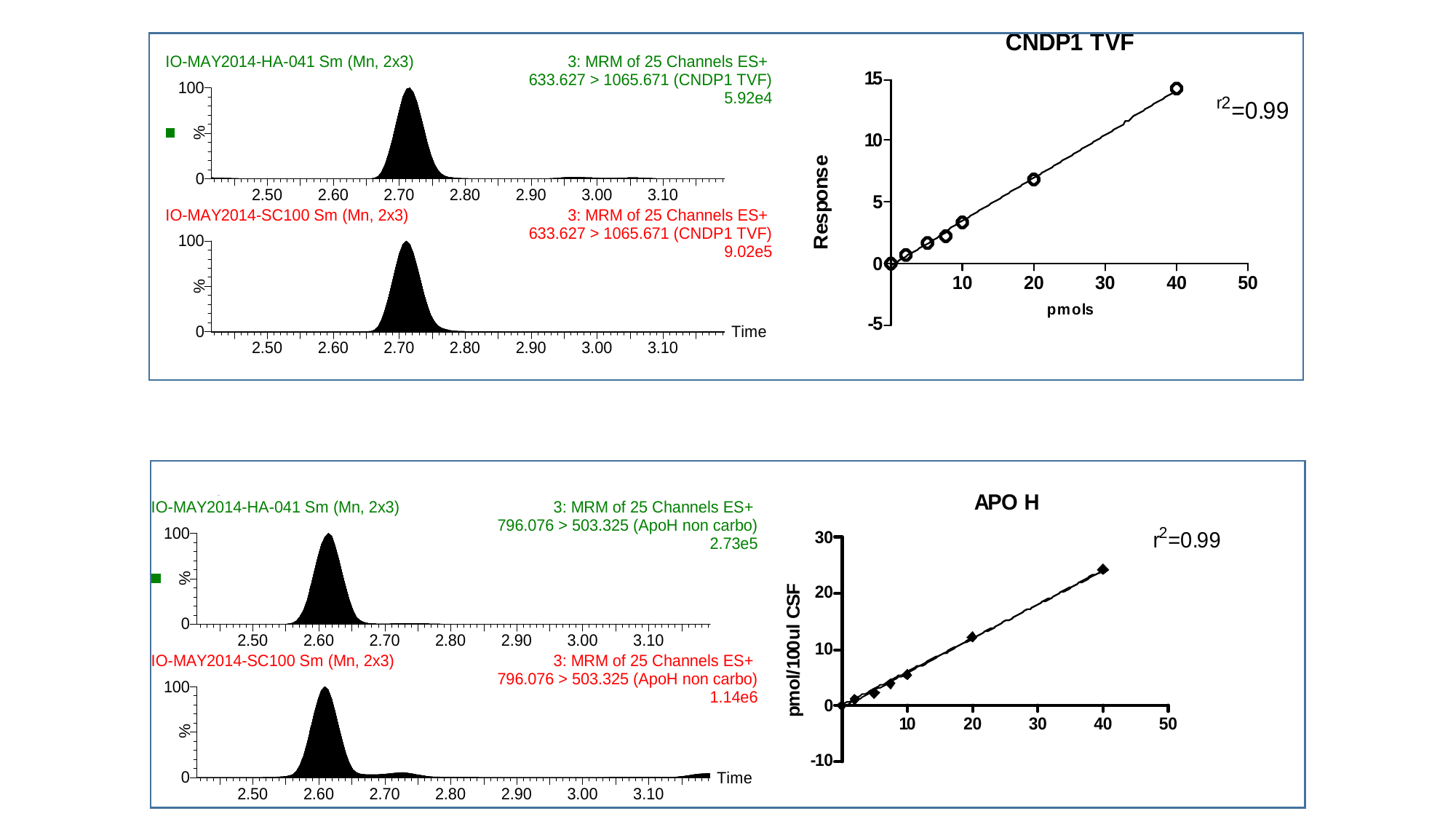

## Slide 5
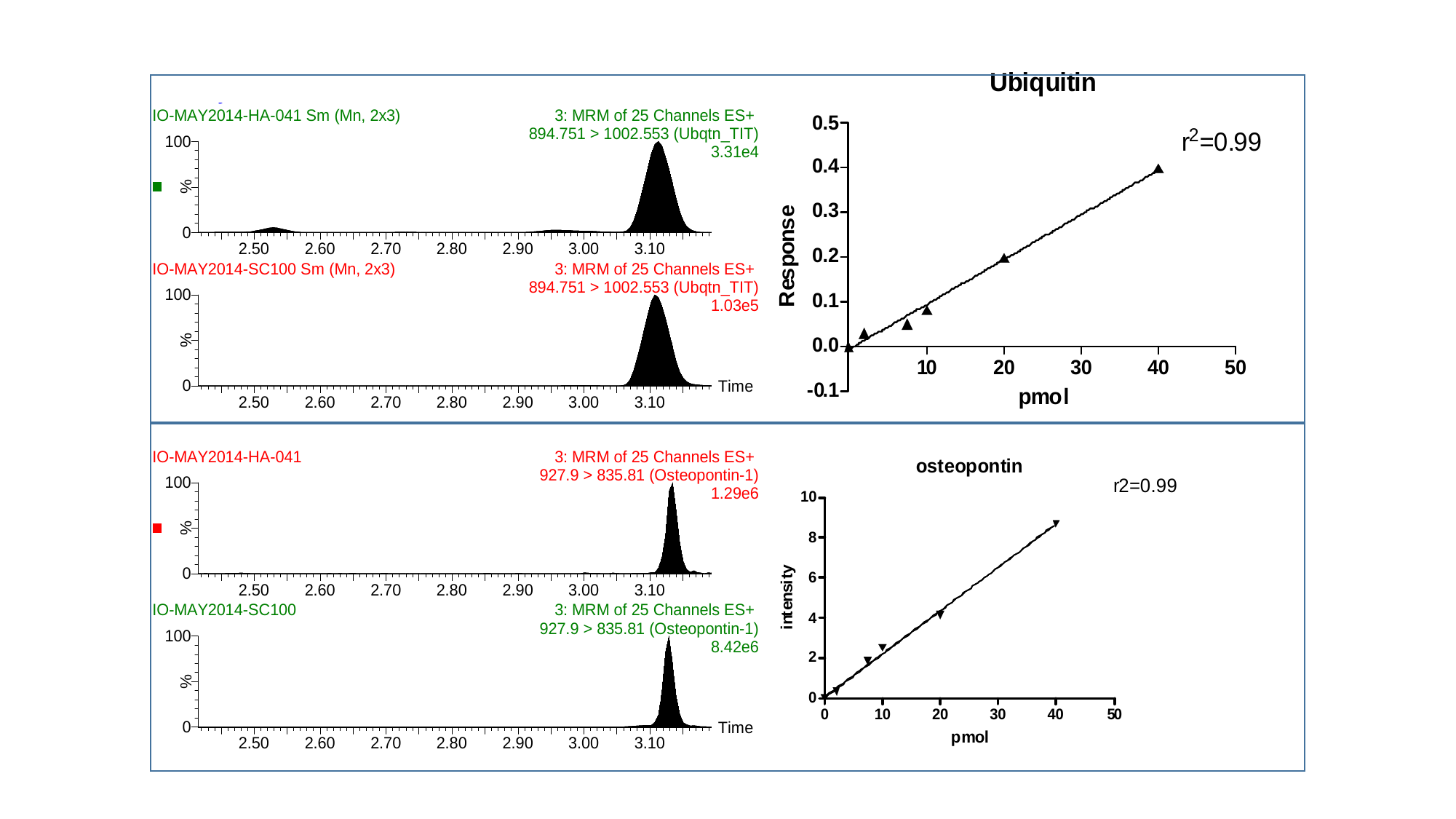

## Slide 6
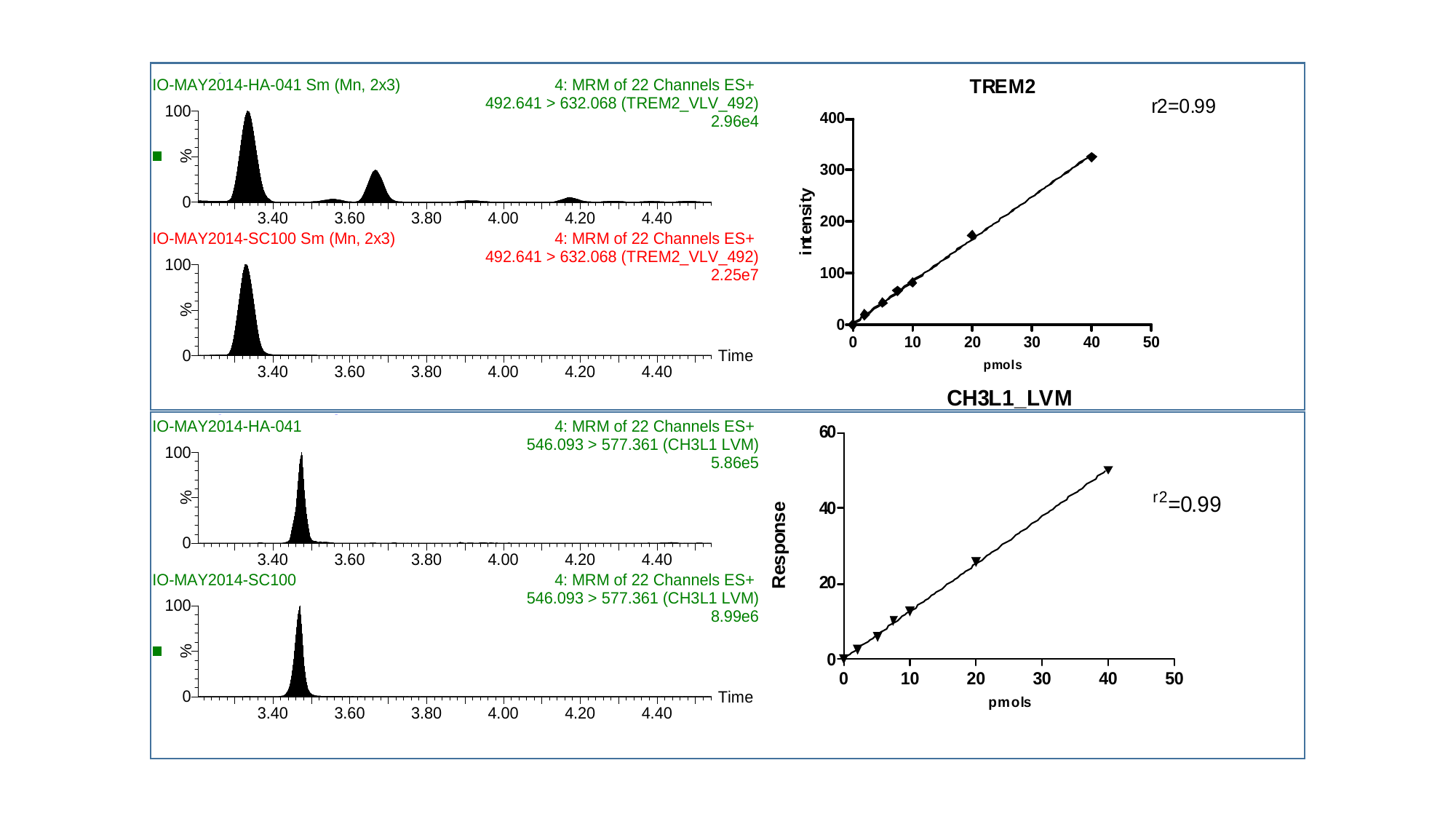

## Slide 7
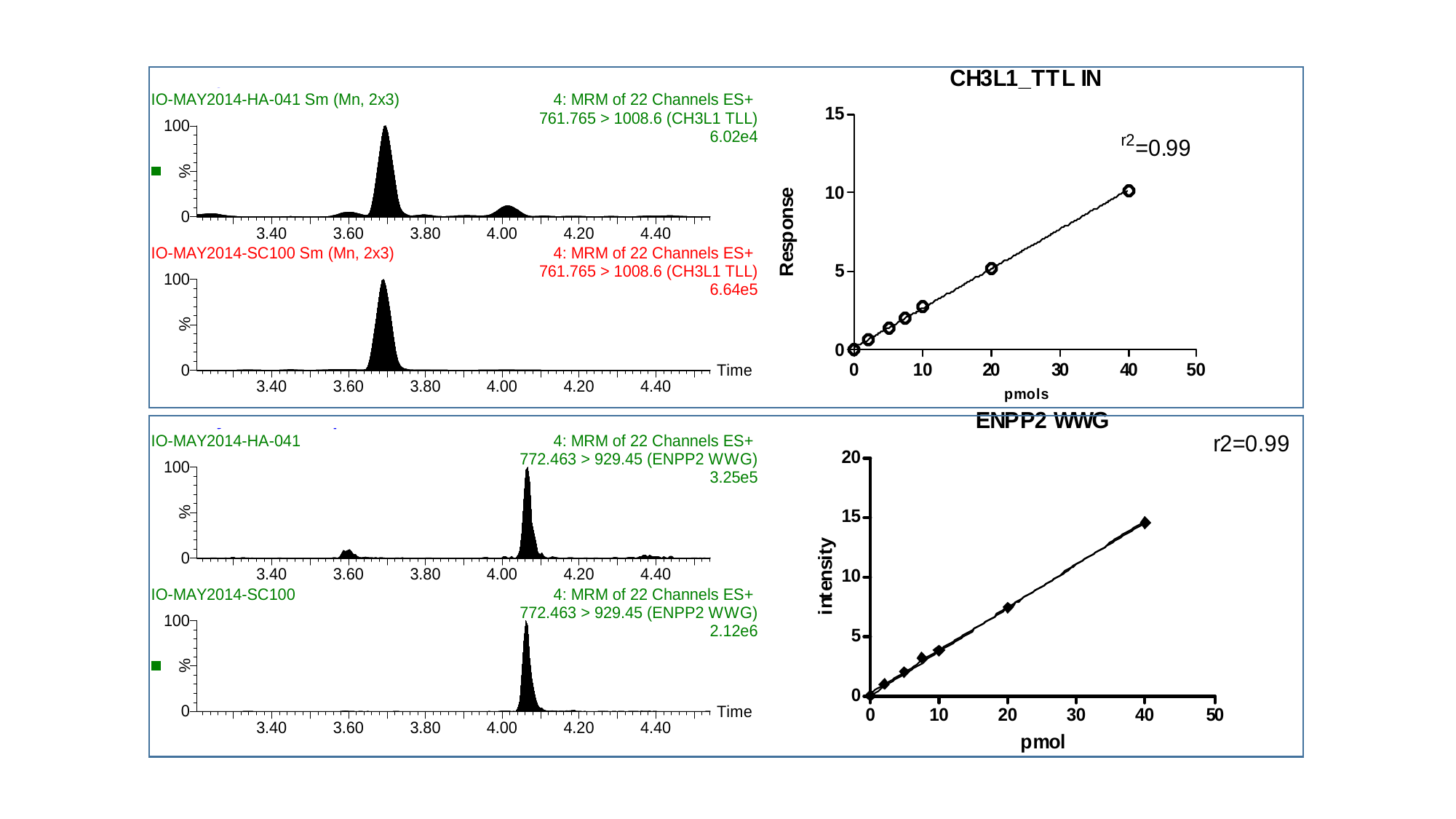

## Slide 8
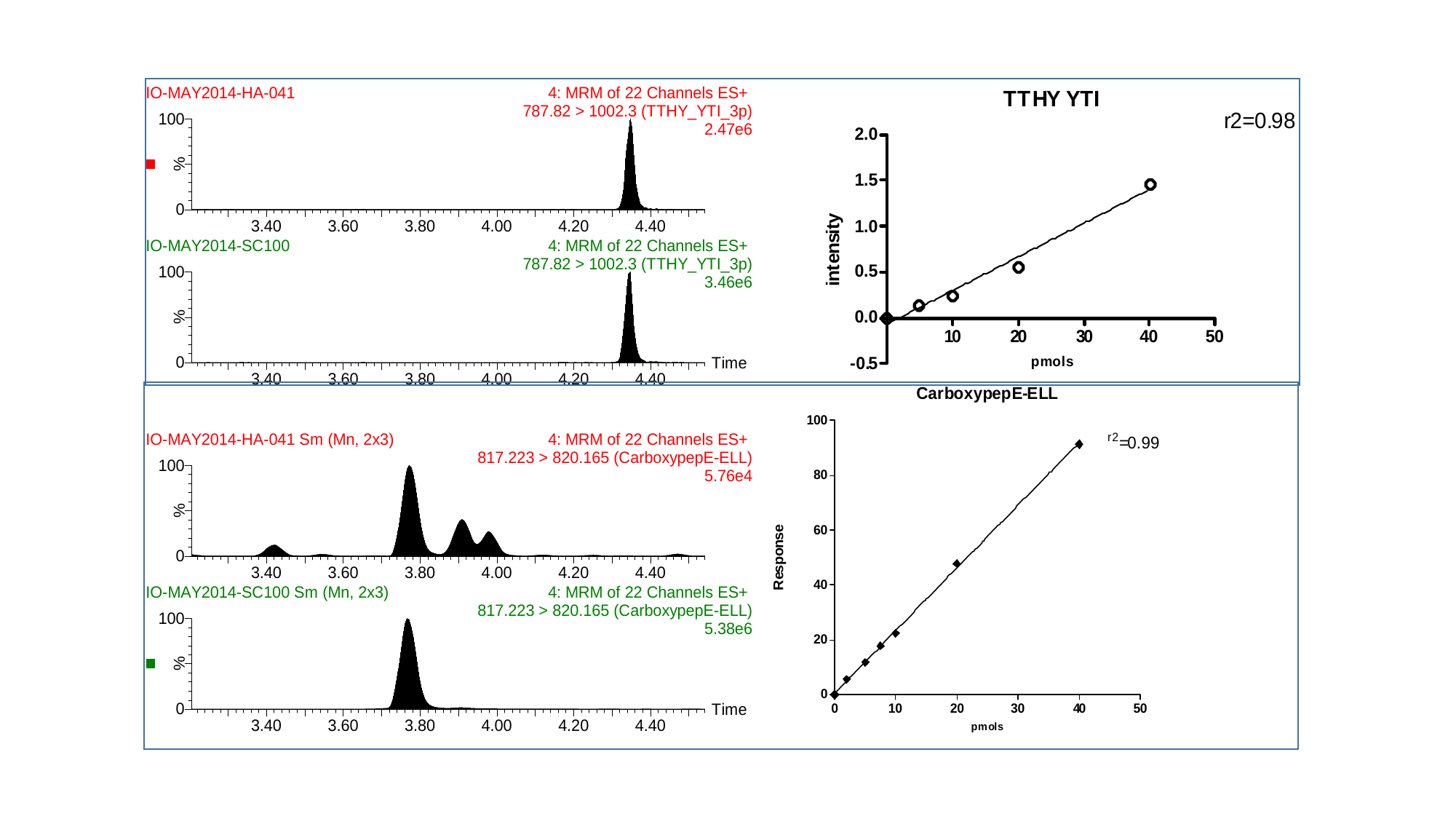

## Slide 9
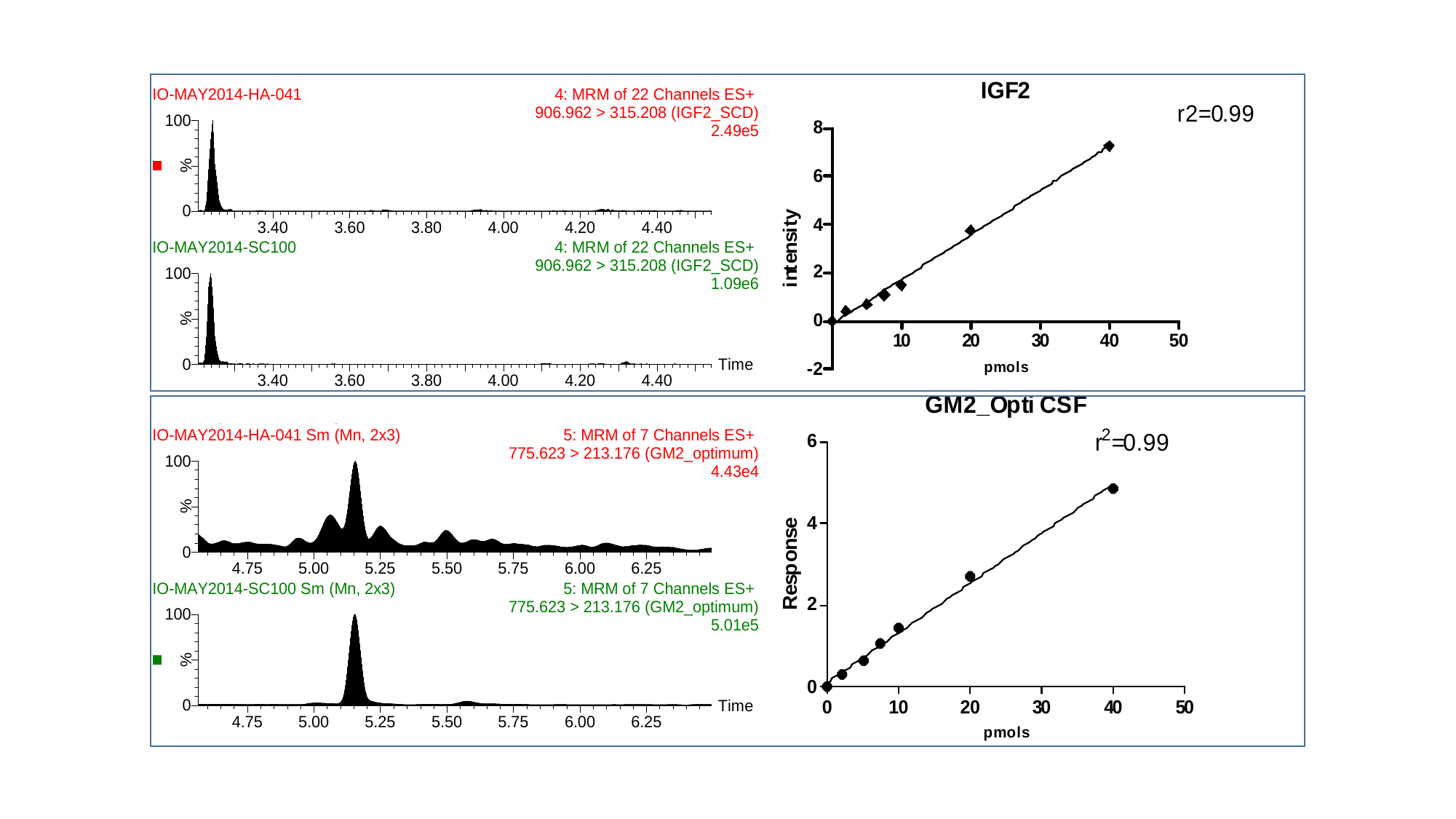

## Slide 10
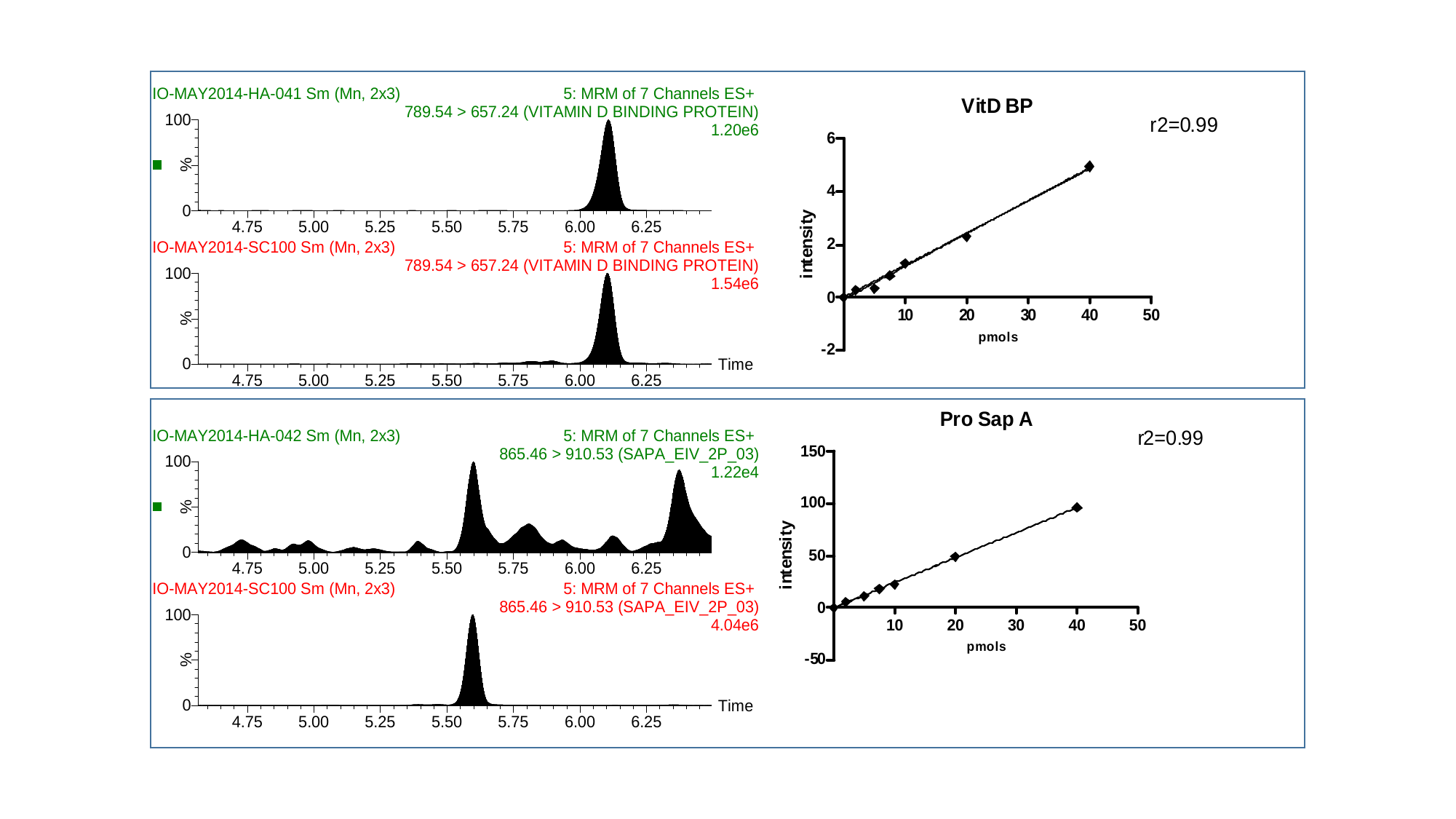

## Slide 11
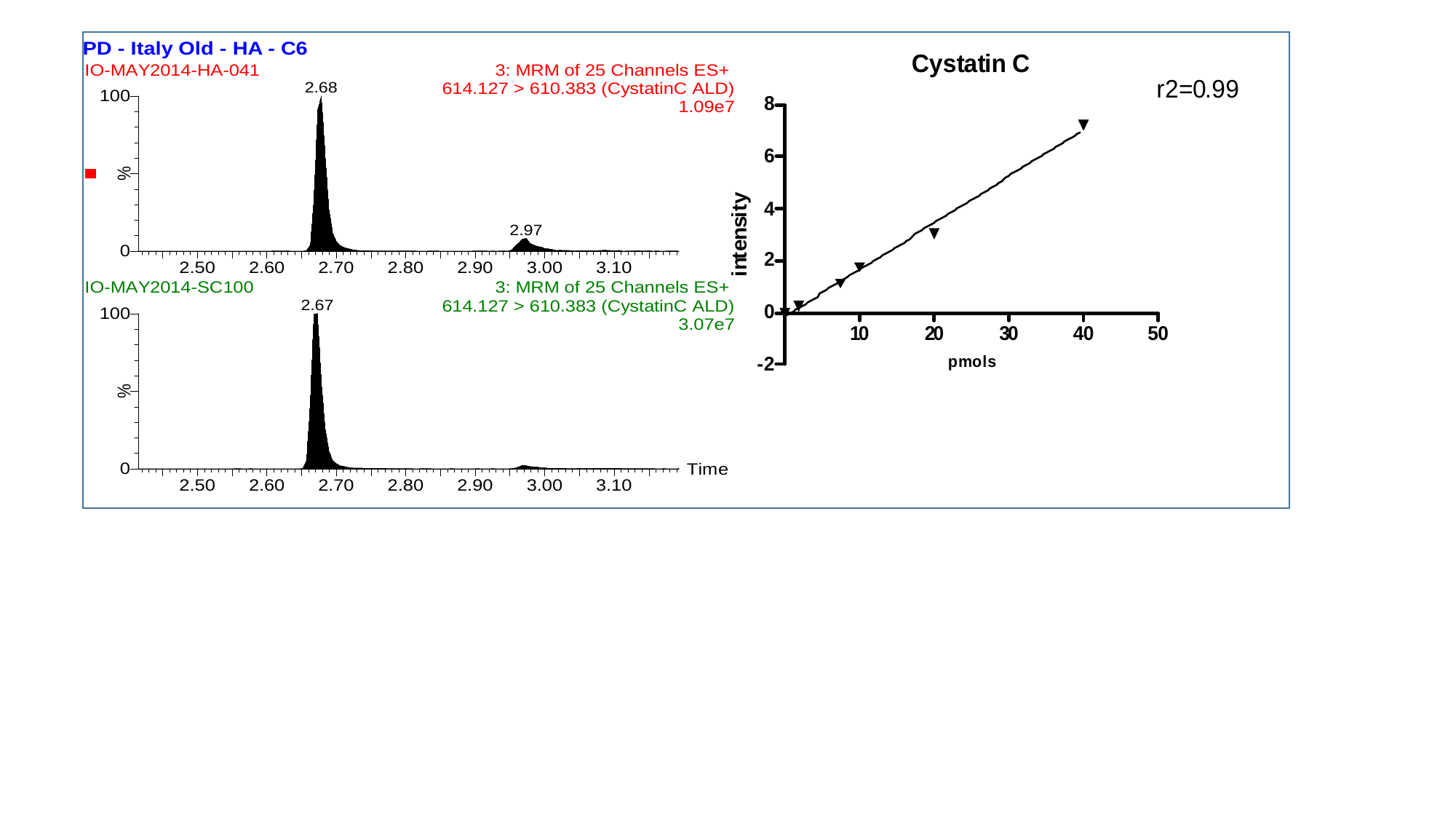

## Slide 12
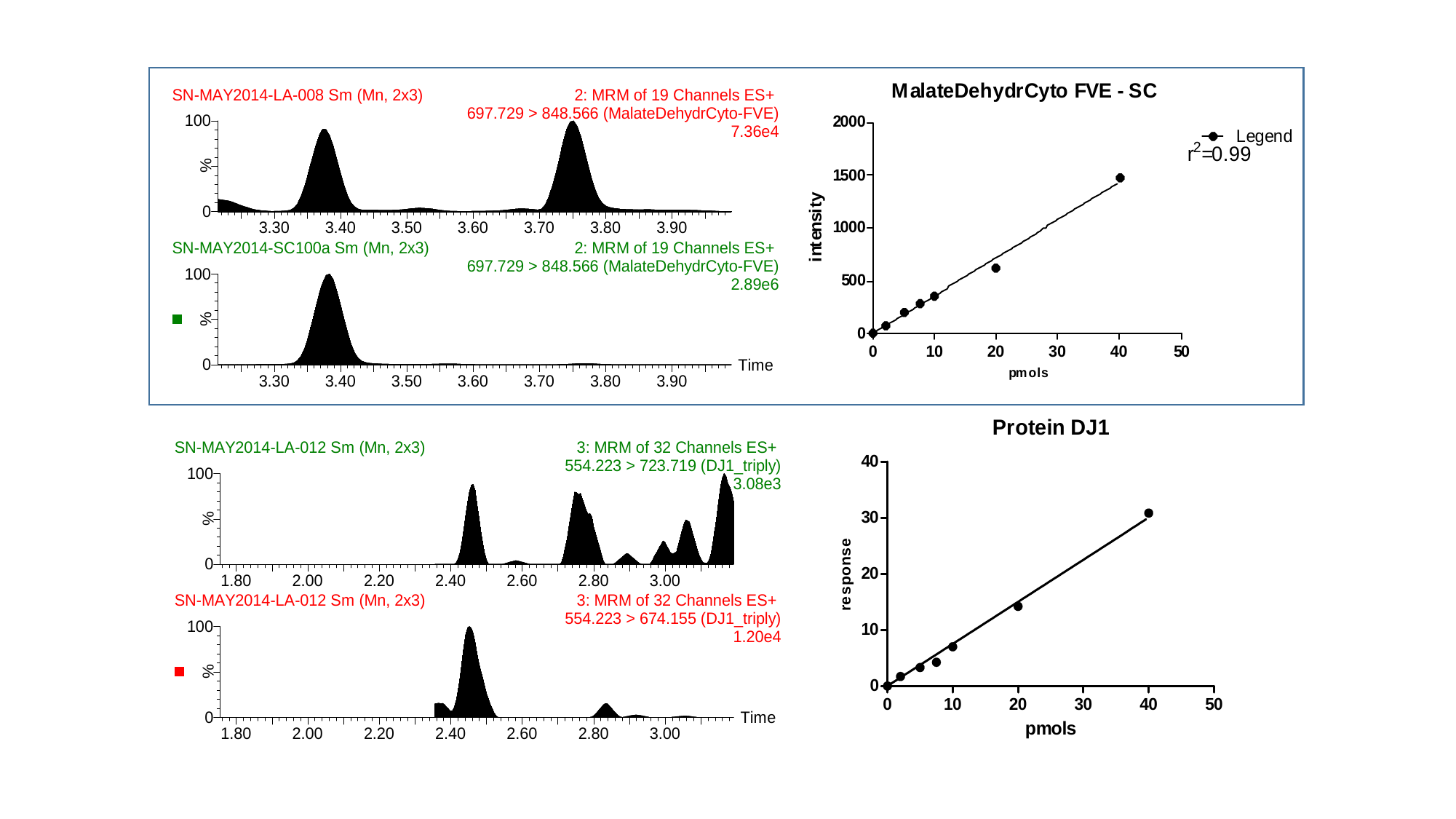

## Slide 13
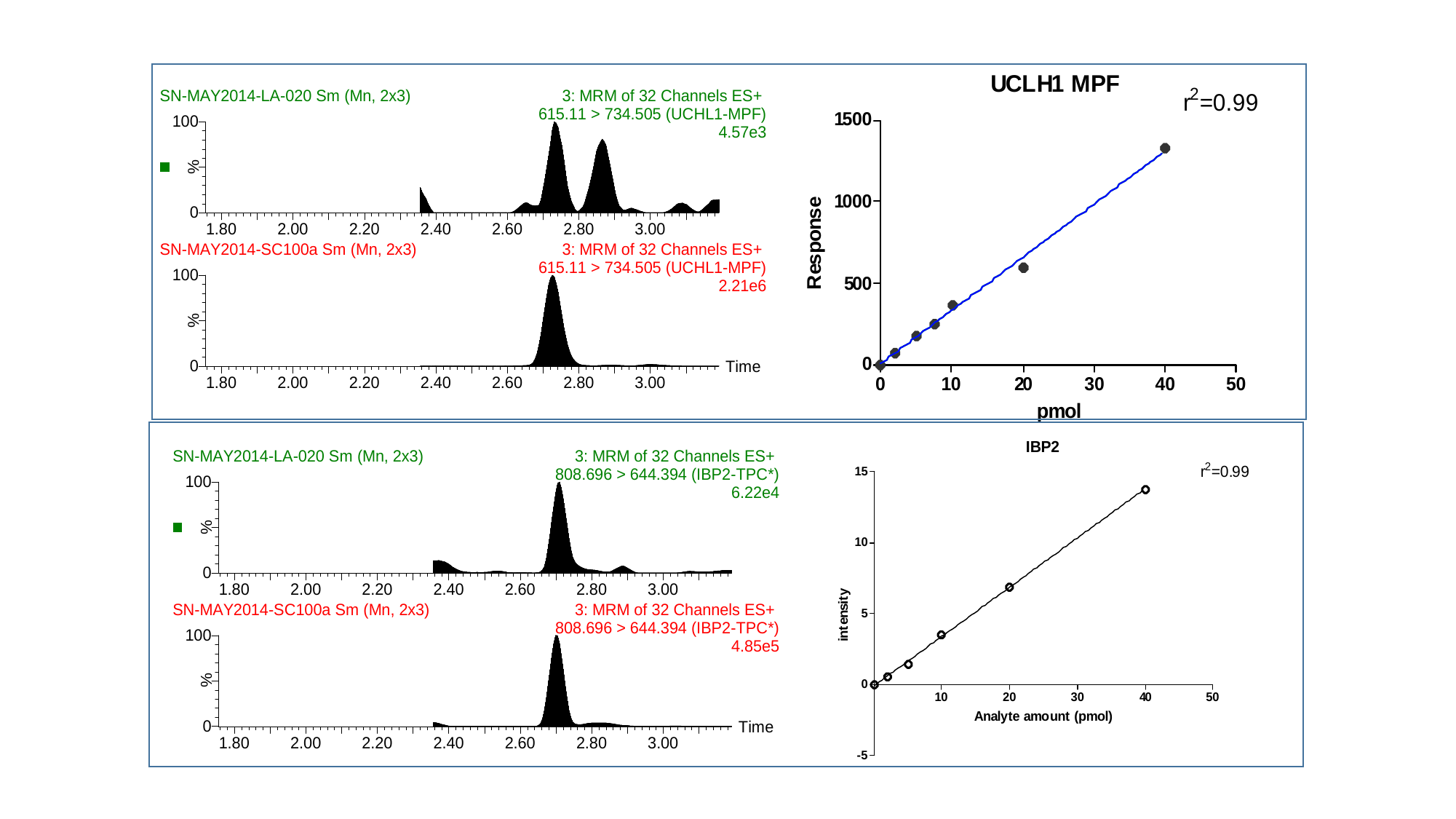

## Slide 14
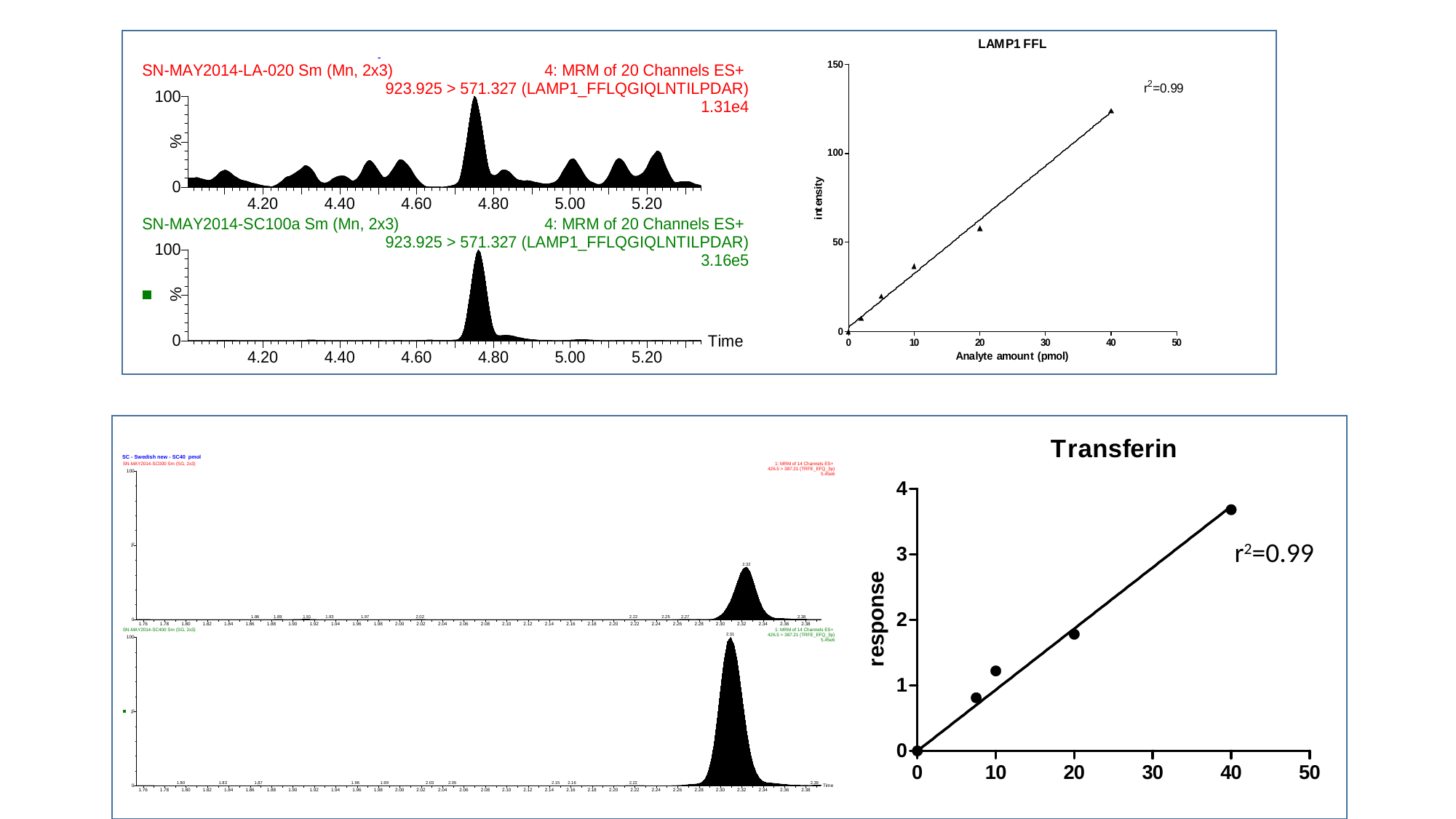

r2=0.99

## Slide 15
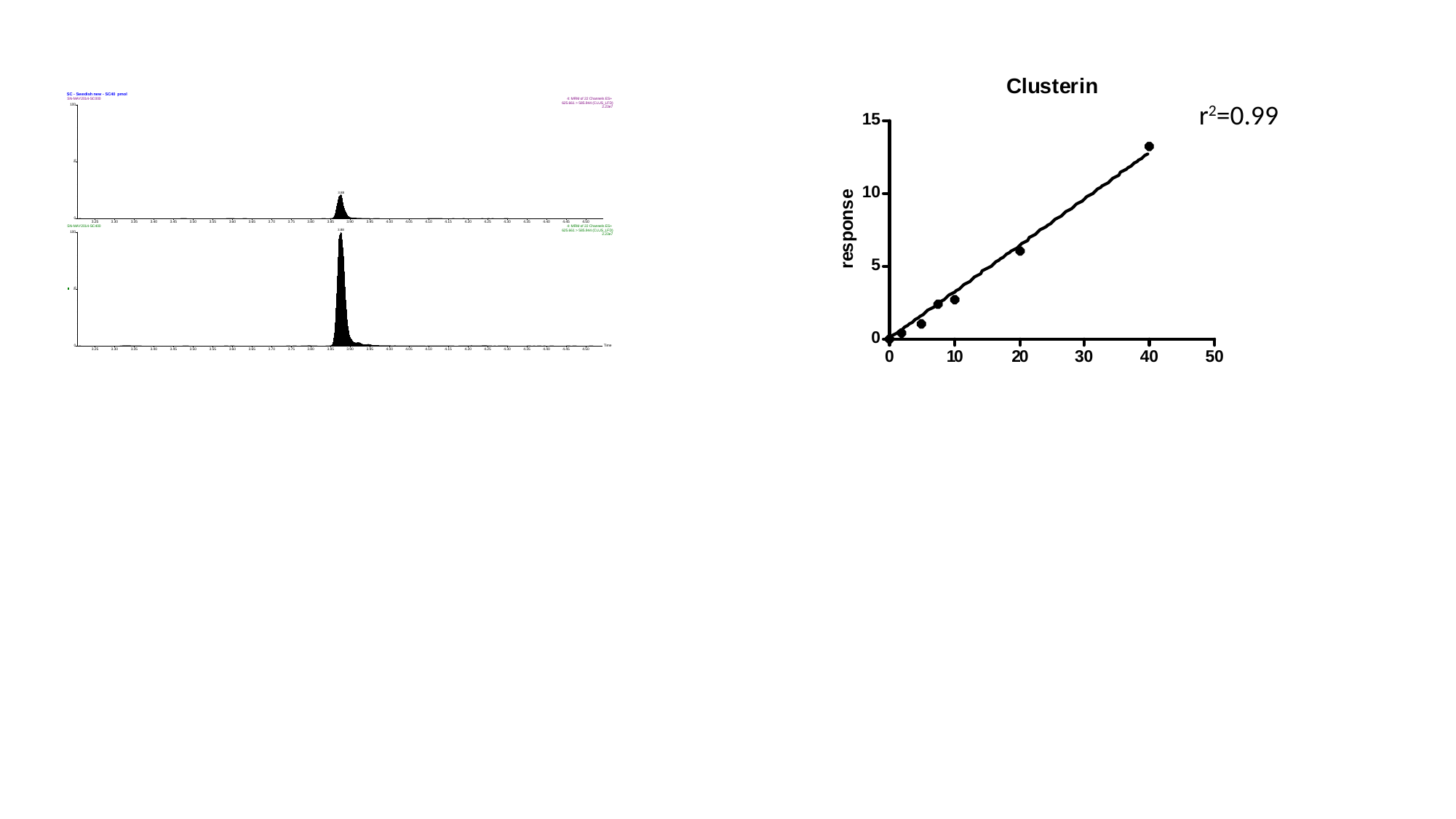

r2=0.99
